# Zwitterionic lipid nanoparticles for efficient siRNA delivery and hypercholesterolemia therapy with rational charge self-transformation

**DOI:** 10.7150/thno.111685

**Published:** 2025-03-01

**Authors:** Ruichen Zhao, Jing Guo, Ziqi Liu, Yusheng Zhang, Jiamin Zuo, Songzhang Lv, Xianyu Li, Wenlong Yao, Xin Zhang

**Affiliations:** 1 State Key Laboratory of Biopharmaceutical Preparation and Delivery, Chinese Academy of Sciences, Beijing, 100190, PR China.; 2 State Key Laboratory of Natural and Biomimetic Drugs, School of Pharmaceutical Sciences, and Chemical Biology Center, Peking University, Beijing, 100191, PR China.; 3 Beijing Key Laboratory of Traditional Chinese Medicine Basic Research on Prevention and Treatment for Major Diseases, Experimental Research Center, China Academy of Chinese Medical Sciences, Beijing, 100700, PR China.; 4 School of Chemical Engineering, University of Chinese Academy of Sciences, Beijing, 100049, PR China.

**Keywords:** Zwitterionic lipid nanoparticles, efficient siRNA delivery, hypercholesterolemia, PCSK9, rational charge self-transformation

## Abstract

**Rationale:** Effective delivery of small interfering RNA (siRNA) remains a significant challenge in treating hypercholesterolemia due to biocompatibility, cellular uptake, and endosomal escape issues. Rational regulation of carrier surface charge contributes to efficient siRNA delivery *in vivo*.

**Methods:** This study introduces zwitterionic lipid nanoparticles (ZwiLNPs) as a novel solution to these challenges. By leveraging the unique properties of zwitterionic polymers, we achieved robust siRNA encapsulation and targeted delivery. The design of ZwiLNPs facilitates charge self-transformation in response to physiological conditions, which enhances their biocompatibility and cellular uptake efficiency.

**Result:** *In vivo* studies demonstrated significant liver-targeting capabilities of ZwiLNPs, with improved endosomal escape following cellular internalization. Comparative analyses confirmed that ZwiLNPs outperform conventional lipid nanoparticles in terms of both cellular uptake and endosomal release.

**Conclusion:** These findings position ZwiLNPs as a promising platform for RNA interference therapies, particularly for hypercholesterolemia and other lipid-related disorders.

## Introduction

Small interfering RNA (siRNA) therapy represents a significant advancement in treating hypercholesterolemia and other chronic diseases 1-6. By specifically targeting and silencing mRNA of key genes, such as proprotein convertase subtilisin/kexin type 9 (PCSK9), involved in lipid metabolism, siRNA can achieve precise reductions in low-density lipoprotein (LDL) levels, effectively addressing hypercholesterolemia at a molecular level. This targeted approach minimizes off-target effects and reduces the risk of adverse reactions, providing a more favorable safety profile than traditional pharmacological therapies.

The efficacy of siRNA therapeutics depends on delivery technology, which is essential for transporting siRNA molecules to target tissues. Due to their inherent instability, unmodified siRNA requires advanced delivery systems to enhance bioavailability and ensure cellular uptake 7-10. Lipid nanoparticles (LNPs) have emerged as a promising approach, offering advantages in encapsulation efficiency and reduced immunogenicity. Effective delivery protects siRNA from degradation and optimizes tissue localization, ultimately enhancing therapeutic outcomes for hypercholesterolemia and other chronic diseases 11-15. Continued innovation in delivery technologies is essential for advancing siRNA therapies into clinical practice.

LNPs delivering siRNA can be refined into multiple biological processes, each with distinct property requirements. During the siRNA loading phase, LNPs typically require a stronger positive charge to enhance their interaction with siRNA 16-18. However, in the bloodstream, LNPs must maintain a neutral charge to prevent non-specific protein adsorption, which can lead to clearance and toxicity 19. LNPs naturally target the liver as an RNA drug delivery system 20-22. However, a stronger positive charge is necessary on their surface during endosomal escape within hepatocytes. This elevated positive charge facilitates electrostatic interactions with anionic components of the endosomal membrane, promoting LNP degradation and enabling siRNA release into the cytoplasm for effective RNA interference (RNAi) 23-26. Consequently, the modulation of charge throughout different delivery processes significantly influences the efficiency of siRNA delivery by LNPs.

Additionally, while the zwitterionic nature of the ZwiLNPs has shown beneficial properties, charge regulation presents significant challenges. Charge interactions between nanoparticles and cellular membranes heavily influence the delivery of mRNA and siRNA. The balance is delicate; while cationic lipids can enhance membrane fusion and uptake, excess positive charge can lead to cytotoxic effects and provoke unwanted immune responses. Although we have improved endosomal escape through the proton sponge effect, further optimization in charge tuning remains essential. This necessitates ongoing investigation into the physicochemical properties of LNPs in various biological contexts. Understanding how factors such as pH, ionic strength, and cellular environment influence the charging behavior of LNPs will substantially improve the development of formulations that can navigate physiological barriers more effectively.

**Scheme 1:**
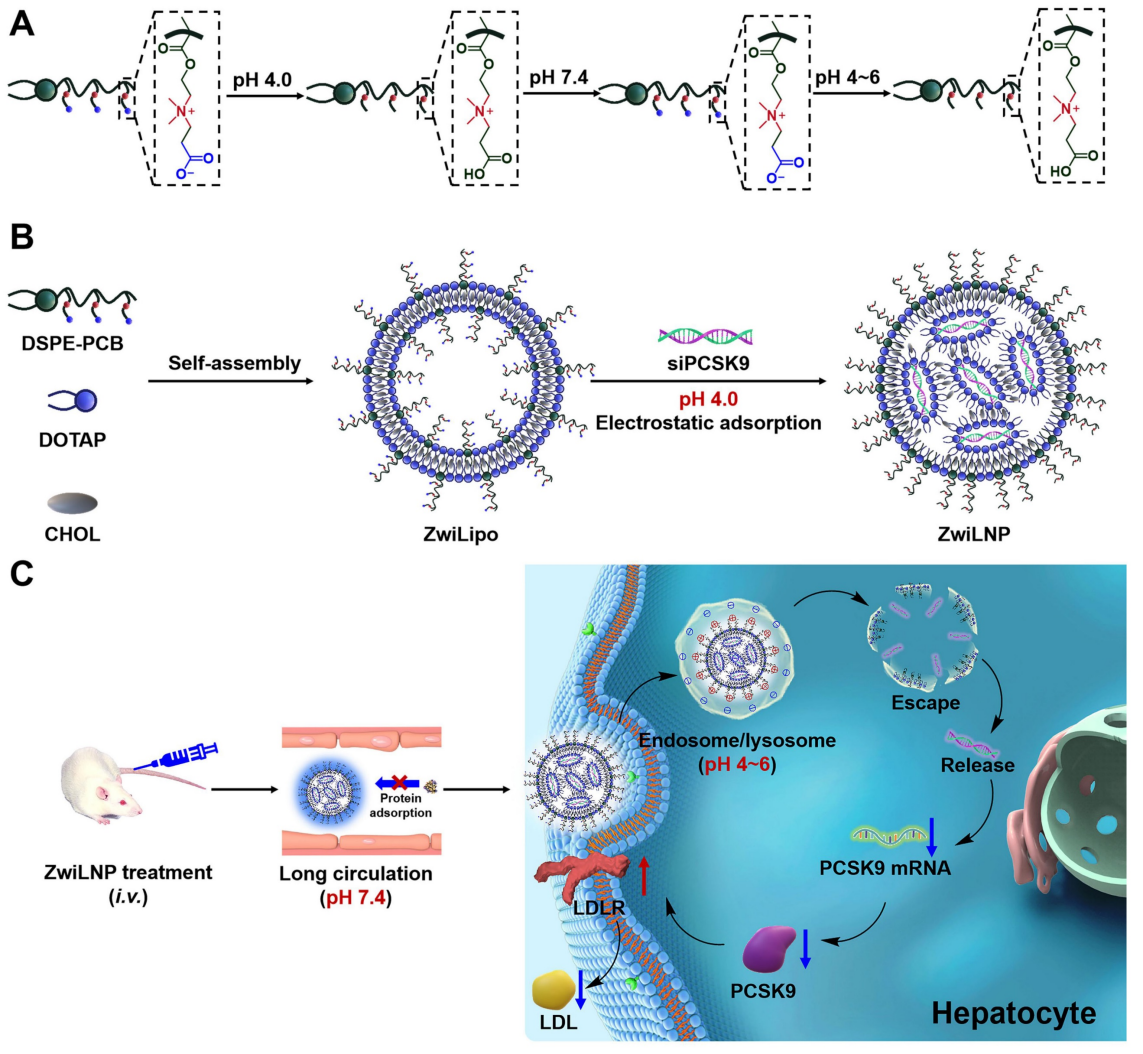
The composition and mechanism of ZwiLNPs for in vivo delivery. (A) Scheme of rational charge self-transformation of zwitterionic polymers. Under acidic conditions (pH = 4), the zwitterionic polymer transforms into a cationic polymer by protonating its anionic groups. At near-neutral physiological conditions (pH=7.4), the protonated carboxyl groups rapidly deprotonate, restoring the zwitterionic polymer to an electric-neutral state. (B) The composition of zwitterionic LNPs (ZwiLNPs). (C) Scheme of the in vivo delivery of ZwiLNPs and the treatment mechanism for hypercholesterolemia. After intravenous administration, ZwiLNPs achieve long-circulating properties due to their anti-protein adsorption characteristics. Upon reaching the target organ and being taken up by cells, ZwiLNPs utilize the proton sponge effect of the zwitterionic polymer for endosomal escape, releasing siPCSK9 into the cytosol to exert therapeutic effects.

In this study, we introduced a zwitterionic polymer (DSPE-PCB) at the terminal of lipid molecules to enhance the efficient delivery of siRNA via LNPs. As shown in Scheme 1A, the zwitterionic polymer features a repeating unit with a pair of oppositely charged groups. Under acidic conditions, the zwitterionic polymer transforms into a cationic polymer by protonating its anionic groups. At near-neutral physiological conditions, the protonated carboxyl groups rapidly deprotonate, restoring the zwitterionic polymer to an electric-neutral state. This proton buffering mechanism allows for a faster and more sensitive molecular charge transition than charge transitions based on chemical bond cleavage or structural changes. Importantly, this charge transition is reversible; when the zwitterionic polymer is exposed to acidic conditions again, the anionic carboxylate groups undergo protonation, resulting in a charge transition 27-32.

Based on the proton buffering-mediated charge transition mechanism, lipid nanoparticles containing zwitterionic polymers (ZwiLipo) can efficiently encapsulate siRNA at pH 4.0, leading to the formation of zwitterionic LNPs (ZwiLNPs) (Scheme 1B). ZwiLNPs rapidly transition to a neutral charge during circulation, resisting non-specific protein adsorption through their hydrated surface, thereby achieving prolonged circulation time 33. Upon hepatocyte uptake, ZwiLNPs again convert to cationic LNPs in endosomes/lysosomes via protonation, facilitating endosomal escape and releasing siRNA for RNAi therapy targeting hypercholesterolemia (Scheme 1C).

## Results

### The characterization of zwitterionic polymer, liposomes, and LNPs

**Figure 1:**
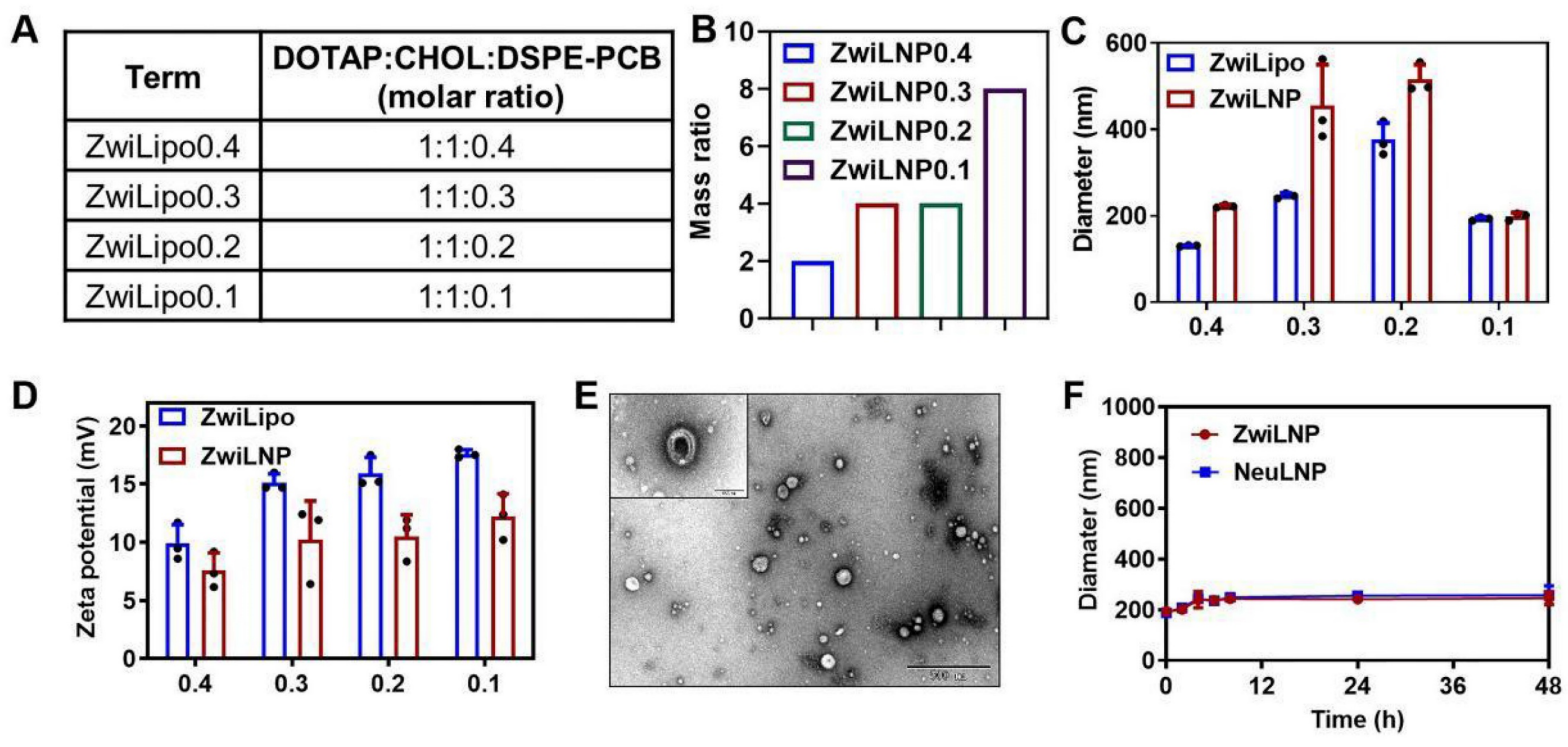
The characterization of zwitterionic polymer, liposomes, and LNPs. (A) The molar ratio of zwitterionic LNPs. (B) The mass ratio of ZwiLNPs: siRNA. (C, D) The diameter and zeta potential of ZwiLipo and ZwiLNPs. (E) The TEM image of ZwiLNPs. Scale bar: 500 nm. The inset is an enlarged TEM image. Scale bar: 100 nm. (F) The serum stability of ZwiLNP and NeuLNP. (The molar ratio of ZwiLNP: DOTAP:CHOL:DSPE-PCB = 1:1:0.1; the molar ratio of NeuLNP: DOTAP:CHOL:DSPE-PEG = 1:1:0.1) Data were presented as the mean ± SD (n = 3).

The zwitterionic polymer DSPE-PCB, positioned at the terminus of lipid molecules, was synthesized using a novel approach that involves the polymerization of zwitterionic monomers initiated by lipid molecules, as reported by our laboratory (Figures S1-S6) 34, 35. This method enables precise control over polymer length and structure, facilitating the design of polymers with tailored biomedical properties. Following its synthesis, DSPE-PCB was incorporated into the formulation of lipid nanoparticles, referred to as ZwiLipo, at varying ratios to evaluate its impact on siRNA loading efficiency (Figure 1A).

As the content of DSPE-PCB in the formulation increased, we observed a marked enhancement in the efficiency of siRNA loading into ZwiLipo. This observation suggests that the zwitterionic properties of DSPE-PCB facilitate better interaction with the siRNA molecules, ultimately leading to more efficient encapsulation (Figures S7 and 1B). Notably, formulations designated as ZwiLipo0.2 and ZwiLipo0.3 exhibited larger particle sizes compared to their counterparts, and this size further increased following the loading of siRNA (Figure 1C). Such a phenomenon may be attributed to the incorporation of siRNA molecules into the lipid matrix, resulting in a more complex nanoparticle structure.

At a pH of 4.0, ZwiLipo demonstrated a high positive charge, which is advantageous for promoting interactions with negatively charged biomolecules, such as nucleic acids. After siRNA loading, the zeta potential of the resulting ZwiLNP formulations decreased yet maintained a positive charge overall (Figure 1D).

Transmission electron microscopy (TEM) analysis revealed that ZwiLNP appeared spherical in morphology yet, intriguingly, was significantly smaller than the dimensions measured by dynamic light scattering (DLS). This discrepancy is likely a result of the hydration layer formed around the ZwiLNP surface, which may influence the hydrodynamic behavior observed in DLS measurements (Figures 1E, S8 and S9). The presence of this protective hydrated layer is crucial as it not only stabilizes the nanoparticles in physiological conditions but also shields the encapsulated siRNA from enzymatic degradation. Benefiting from this protective hydrated layer, ZwiLNP exhibited excellent serum stability (Figure 1F). We also evaluated the encapsulation efficiency of different ZwiLNP formulations for siRNA, with all ZwiLNP demonstrating high efficiency (Figure S10). Leveraging the advantages provided by this protective hydrated layer, ZwiLNP exhibited remarkable serum stability, crucial for maintaining therapeutic efficacy in biological environments (Figure 1F). The excellent stability not only prolongs circulation time in the bloodstream but also minimizes premature degradation of the siRNA payload. In addition, we synthesized neutral polymers at the terminus of lipid molecules, specifically 1, 2-distearoyl-sn-glycero-3-phosphoethanolamine-N-[methoxy (polyethylene glycol)-2000] (DSPE-PEG), to create siRNA-loaded lipid nanoparticles (NeuLNPs) (Figure S11). These NeuLNPs also demonstrated outstanding serum stability.

### The hepatocyte-target of ZwiLNPs

**Figure 2:**
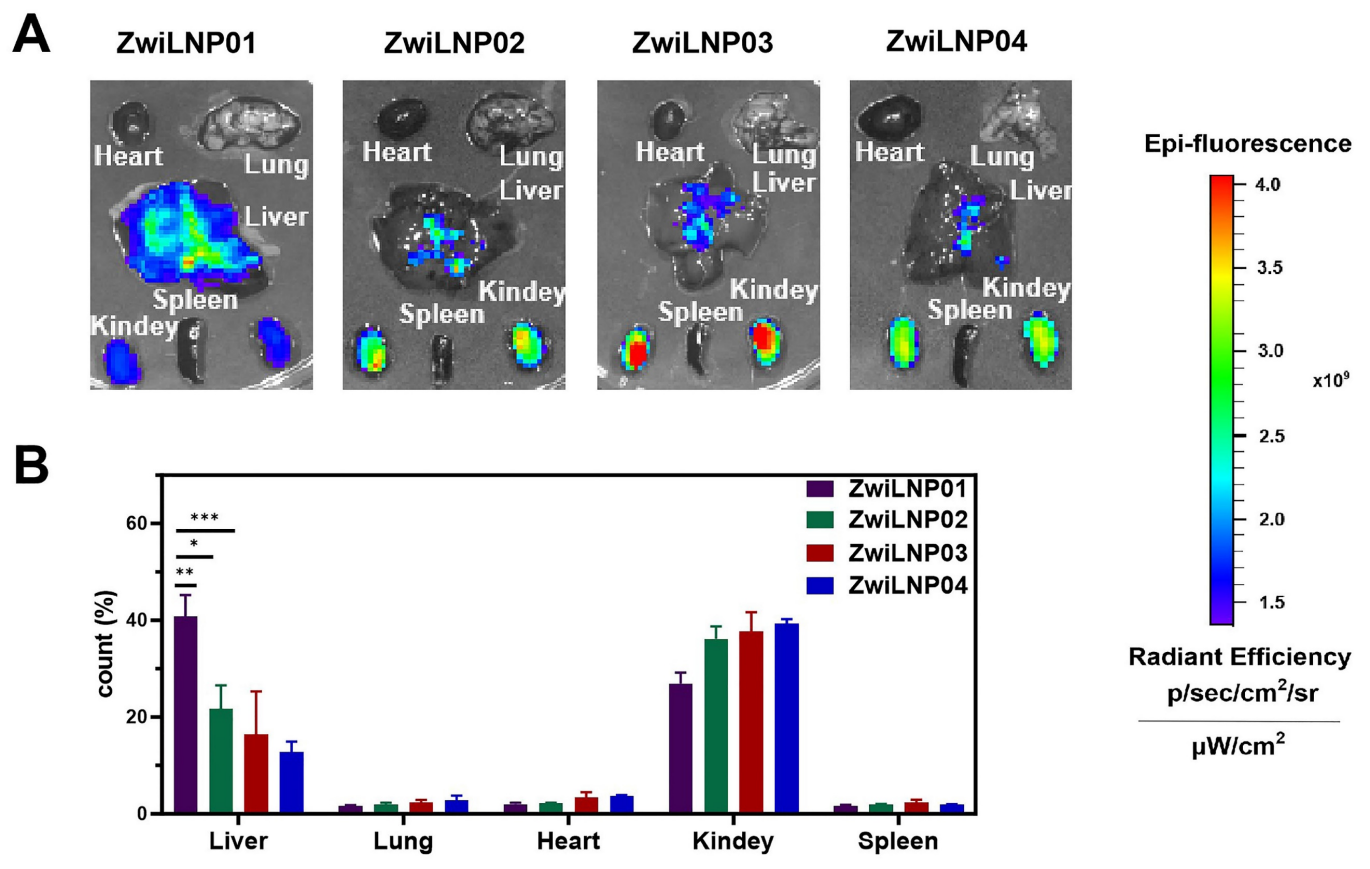
The screening for hepatocyte-target capability of ZwiLNPs. (A) The fluorescence image of the biodistribution of ZwiLNPs. (B) The fluorescence count of liver, lung, heart, kidney, and spleen after ZwiLNP treatment. Data were presented as the mean ± SD (n = 3).

We evaluated the effect of varying concentrations of zwitterionic polymer on the liver-targeting efficiency of LNPs. Our findings, illustrated in Figure 2A, indicate that ZwiLNP0.1 exhibited the strongest drug fluorescence signal in the liver, highlighting its potential as an effective delivery system for RNA-based therapeutics. Interestingly, while ZwiLNP0.1 demonstrated robust liver targeting, the fluorescence signal of siRNA in the kidneys was also notably high. This raises important questions regarding the biodistribution and pharmacokinetics of the formulations, as efficient targeting of the liver is often desired for therapeutic efficacy, particularly in the context of diseases like hypercholesterolemia.

In contrast to ZwiLNP0.1, the other ZwiLNP formulations exhibited significantly weaker siRNA signals in the kidneys. This observation suggests that alterations in the zwitterionic polymer concentration can have pronounced effects on organ-specific distribution. To further investigate these dynamics, we quantified the distribution of siRNA fluorescence signals across various organs. As depicted in Figure 2B, our analysis revealed no significant differences in siRNA fluorescence signals in both the liver and kidneys among the ZwiLNP formulations. This suggests a threshold effect whereby the molar ratio of the zwitterionic polymer may stabilize the delivery system without significantly altering its distribution profile.

These results indicate that when the molar ratio of DSPE-PCB varies from 9.1% (ZwiLNP0.2) to 16.7% (ZwiLNP0.4), the content of the zwitterionic polymer does not significantly affect the *in vivo* distribution of siRNA. Notably, the siRNA delivered by ZwiLNP0.3 displayed a slightly higher percentage of fluorescence signals in the liver than the other ZwiLNPs. This may indicate a more favorable interaction between the liver tissue and ZwiLNP0.3, which warrants further exploration.

Nevertheless, it is crucial to consider that the siRNA signal observed in the kidneys may be attributed to the renal accumulation induced by Cy5 modification, which is known to enhance tissue-specific targeting. Given its promising liver-targeting capabilities, this insight led us to select ZwiLNP0.1 for further studies. Therefore, all references to ZwiLNPs will denote ZwiLNP0.1 in the following text, allowing for a focused discussion on its potential applications in RNAi therapy and other related fields.

### Efficient cellular uptake and endosomal/lysosomal escape of ZwiLNPs in HepG2

siRNA is a powerful tool for gene silencing, but its inherent properties often hinder its clinical application. Characterized by a high density of negative charges and strong hydrophilicity, siRNA exhibits poor interaction with cell membranes, significantly challenging cellular uptake. The LNP delivery system has emerged as an effective strategy to enhance the delivery of siRNA to target cells. When encapsulated within LNPs, siRNA can be effectively transported to HepG2 cells, a commonly used liver cancer cell line. This effectiveness is attributed mainly to the strong affinity between the lipid bilayer of the LNPs and the cell membrane, facilitating the efficient incorporation of siRNA into the cellular interior.

**Figure 3:**
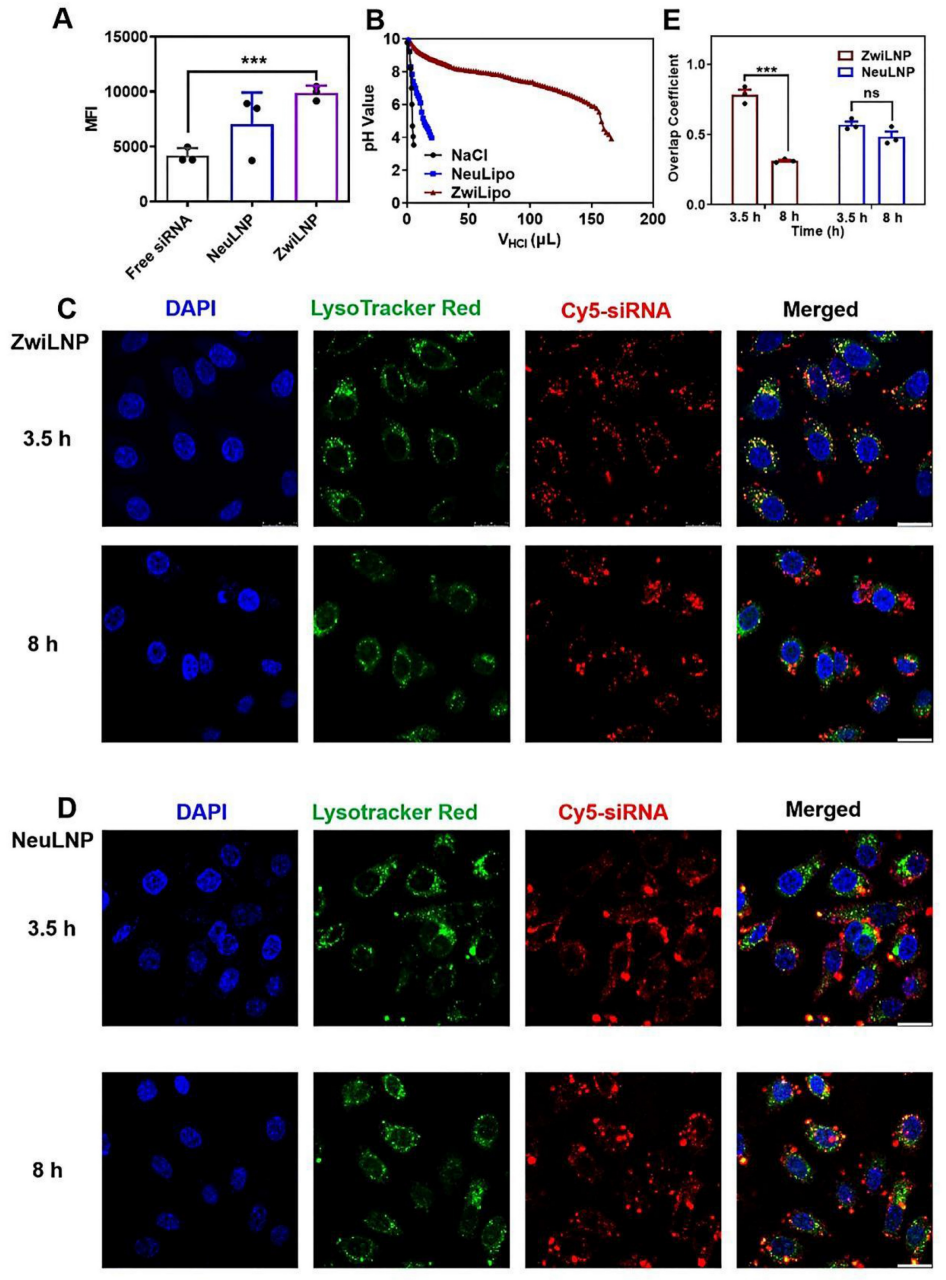
The cellular uptake and endosomal/lysosomal escape of ZwiLNPs. (A) The cellular uptake efficiency of ZwiLNP and NeuLNP. (B) The proton buffering capability of ZwiLNP and NeuLNP. (C) Confocal laser scanning microscopy (CLSM) images of HepG2 cells co-cultured with ZwiLNP for 3.5 h and 8 h. (D) CLSM images of HepG2 cells co-cultured with NeuLNP for 3.5 h and 8 h. Cy5-siRNA was shown in red, lysosome was shown in green, and the cell nuclei stained with DAPI was shown in blue. Scale bar: 50 μm. (E) The overlap coefficients of Cy5-siRNA and lysosome were calculated using Image-Pro Plus software. Data were presented as the mean ± SD (n = 3). (t-test, ns p > 0.05, ***p < 0.001).

Among various LNP formulations, ZwiLNPs have demonstrated superior cellular uptake efficiency 31, 32, 36. Their unique composition, enriched with zwitterionic polymers, contributes to their enhanced performance. These zwitterionic polymers improve biocompatibility and modulate interactions with cellular membranes, thereby promoting more efficient endocytosis than conventional formulations, such as NeuLNPs (Figure 3A).

Endosomal and lysosomal escape is a pivotal biological process that governs the intracellular transport of siRNA 37, 38. Once internalized, LNPs containing siRNA are often sequestered within endosomes and lysosomes, which serve as cellular compartments for degradation. The ability of LNPs to facilitate siRNA escape from these compartments is crucial for achieving therapeutic efficacy. Incorporating zwitterionic polymers into ZwiLNPs significantly enhances their ability to mediate endosomal escape through a pronounced proton sponge effect 39, 40. Under the acidic conditions prevalent in endosomes and lysosomes, ZwiLNPs can adsorb many protons. This proton adsorption leads to a positive charge on the ZwiLNPs, which not only facilitates electrostatic interactions with the negatively charged components of the endosomal/lysosomal membrane but also promotes the degradation of the ZwiLNPs, thereby facilitating the release of the siRNA payload.

In contrast, NeuLNPs exhibit minimal proton adsorption under similar conditions (Figure 3B). This lack of interaction limits their ability to mediate effective endosomal escape, thereby reducing the overall therapeutic potential of the siRNA they deliver. Furthermore, the proton adsorption by ZwiLNPs can alter the osmotic pressure across the endosomal/lysosomal membrane, resulting in membrane swelling and accelerating degradation. This phenomenon enhances the release of siRNA into the cytosol, where it can exert its gene-silencing effects.

To evaluate the effectiveness of ZwiLNPs in promoting endosomal escape, we utilized a confocal laser scanning microscope (CLSM) to visualize the co-localization of siRNA with endosomes/lysosomes (Figures 3C-3E). Labeling the endosomal/lysosomal compartments and the siRNA payload with distinct fluorescent markers could assess the extent of siRNA escape over time. At 3.5 hours post-treatment, both ZwiLNPs and NeuLNPs showed significant co-localization with endosomal/lysosomal signals, indicating that both formulations were successfully internalized into these compartments. However, by the 8-hour mark, the co-localization coefficient of siRNA in ZwiLNPs with endosomes/lysosomes significantly decreased. This finding suggests that ZwiLNPs effectively facilitate the escape of siRNA from endosomal/lysosomal compartments, allowing for greater availability of the siRNA in the cytosol for its intended action.

In contrast, the co-localization coefficient of siRNA in NeuLNPs with endosomes/lysosomes exhibited only a slight decrease at 8 hours, and this change was not statistically significant. This observation underscores the limitations of NeuLNPs in facilitating siRNA escape from endosomal compartments. Therefore, ZwiLNPs represent a more adequate formulation for enhancing siRNA's cellular uptake and endosomal escape, maximizing its therapeutic potential.

### 2.4. ZwiLNPs loaded with siPCSK9 enhance half-life in circulation and liver targeting

**Figure 4:**
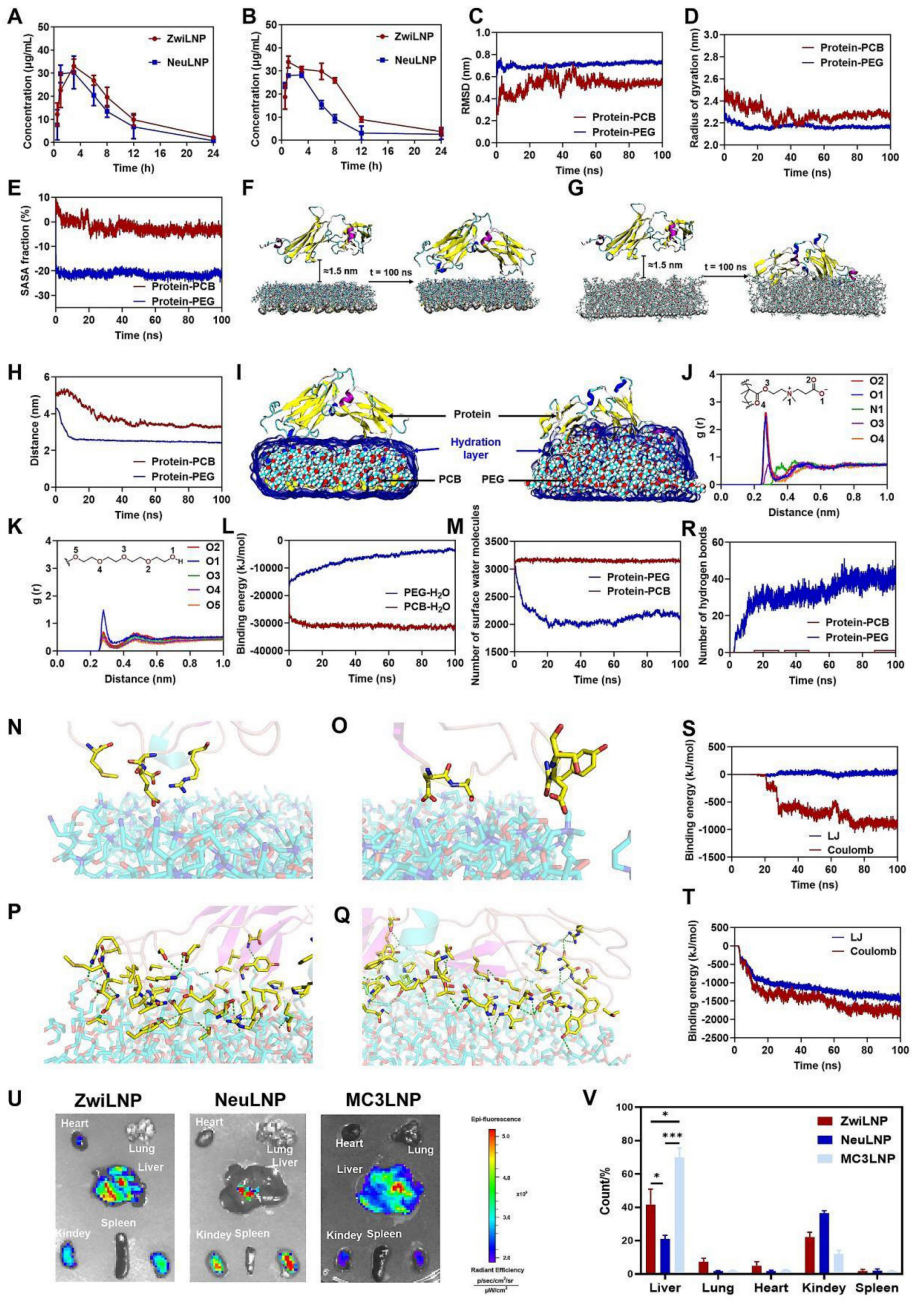
The molecular dynamics simulation of enhancing half-life and liver targeting of ZwiLNPs. (A) The siRNA concentration in the blood after administration of ZwiLNPs and NeuLNPs. (B) The siRNA concentration in the blood after the second administration of ZwiLNPs and NeuLNPs. (C, D) Variation of the RMSD value (C) and the rotational radius (D) of protein in the two systems with simulation time. (E) Variation of the SASA of the two systems with the simulated time. (F, G) The relative positions of protein with PCB (F) and PEG (G) polymers before and after MD simulation. (H) The changes in the distances between the protein and PCB and PEG during MD simulation. (I) The direct interactions between the protein and PCB and PEG. (J) The distance of PCB molecules' O1, O2, and O4 atoms is responsible for hydrogen bonding with water molecules. (K) The interactions in the two systems. (L) The binding energies between the polymer and water molecules. (M) The variation in the number of water molecules at the PCB and PEG surfaces over the simulation time. (N-Q) The binding pattern of protein on the surfaces of PCB (N, O) and PEG (P, Q) polymers (green dashed line indicates hydrogen bonding, yellow stick model shows the amino acid residues in protein that interact with the polymer). (R) Variation of the number of hydrogen bonds between protein, PCB, and PEG polymers in the MD simulation. (S, T) The binding of protein to PCB (S) and PEG (T) in the MD simulation. (U) The biodistribution of ZwiLNPs and NeuLNPs. (V) The fluorescence count of liver, lung, heart, kidney, and spleen spleen with ZwiLNPs and NeuLNPs. Data were presented as the mean ± SD (n = 3). (t-test, *p < 0.05)

Typically, naked siRNA exhibits a half-life of less than 1 hour in circulation, which is inadequate for the therapeutic demands of RNAi 41. Hydrophilic components within LNPs, such as polyethylene glycol (PEG), can enhance circulatory delivery by forming a hydration layer that contributes to a stealthy delivery profile and prolongs the half-life 42. As illustrated in Figure 4A and Figure S12, both ZwiLNPs and NeuLNPs successfully extend the half-life of siRNA to 6.9 hours and 4.9 hours, respectively. While the siRNA concentration in the blood after administration is higher for ZwiLNPs compared to NeuLNPs, it is important to note that the difference in half-life does not reach statistical significance. This result suggests that, although ZwiLNPs may show an improved trend in siRNA delivery in circulation post-initial dosing, there is no significant difference between the two formulations.

Upon initial administration, PEG can elicit the body's immune response, producing anti-PEG protein antibodies. These antibodies can accelerate the rapid clearance of subsequently administered PEGylated LNPs from circulation, a phenomenon known as accelerated blood clearance (ABC) 43. As illustrated in Figure 4B, after the second administration, the half-life of siRNA loaded in NeuLNPs is reduced to 2.8 hours. In contrast, the half-life of siRNA in ZwiLNPs was 10.3 hours, which remains significantly superior to that of NeuLNPs.

The longer half-life of ZwiLNPs and ability to avoid ABC may be attributed to their excellent anti-protein adsorption capability, which is contributed by a dense hydration layer. This hydration layer is closely related to modifying the zwitterionic polymer PCB. Therefore, molecular dynamics (MD) simulations were employed to analyze the interactions between polymers and proteins. Root mean square deviation (RMSD) serves as an important metric for assessing the stability of a system, representing the cumulative deviations of atomic positions from a target conformation at a given moment. Figure 4C illustrates the changes in the RMSD of backbone atoms in the protein-PCB and protein-PEG systems over time. The analysis reveals that during the initial 20 ns, the RMSD values exhibit considerable fluctuations, likely associated with the binding processes between the protein and the surfaces of PCB and PEG. Following 60 ns, the RMSD values stabilize, with average values of 0.539 ± 0.022 nm and 0.725 ± 0.011 nm for the protein-PCB and protein-PEG systems, respectively. The radius of gyration (Rg) describes the overall structural changes; a larger Rg signifies a more expanded state of the system. The variation in Rg over simulation time for both systems is depicted in Figure 4D. Notably, the Rg values for both systems decrease progressively during the first 20 ns of simulation. This reduction can be attributed to the gradual adsorption of the protein onto the surfaces of the PCB and PEG polymers, leading to structural alterations within the protein, resulting in the observed decrease in Rg values. After 60 ns, the Rg values in both systems tend to stabilize, with average values of 2.267 ± 0.022 nm for the protein-PCB system and 2.162 ± 0.008 nm for the protein-PEG system. Analysis of the convergence parameters during the protein adsorption process on PCB and PEG surfaces indicates a more pronounced structural alteration in the protein-PEG system compared to the protein-PCB system. This observation may be attributed to the stronger interactions between the protein and PEG. These findings underscore the advantages of ZwiLNPs in prolonging the half-life of siRNA in circulation and potentially enhancing liver targeting, which is critical for the effectiveness of RNAi therapies aimed at liver-expressed targets.

We further analyzed the interactions between the polymer and protein through molecular dynamics simulations. Prior to the simulations, the initial structures of the zwitterionic polymer PCB and the neutral polymer PEG were constructed separately (Figure S13). They were then simulated in an aqueous solution for 20 ns to reach a stable state. To analyze the structural changes of the system during the simulation, we examined the solvent-accessible surface area (SASA) of the protein in both the protein-PCB and protein-PEG systems throughout the MD simulation (Figure 4E). In the protein-PCB system, the SASA value of the protein gradually decreased during the first 40 ns of the MD simulation. It stabilized after 60 ns, resulting in an approximate reduction of 5%. In contrast, the SASA value in the protein-PEG system exhibited a rapid decrease during the initial simulation phase, indicating that the protein quickly adsorbed onto the surface of the PEG polymer. Overall, the SASA value of the protein decreased by approximately 20%.

To investigate the protein adsorption process on the polymer surface, we extracted the structures before and after the MD simulation for analysis (Figures 4F and 4G). In the protein-PCB system, after 100 ns of MD simulation, the protein was primarily adsorbed at both ends onto the PCB polymer surface, with relatively few amino acids interacting. Conversely, in the protein-PEG system, the protein had partially embedded into the PEG polymer surface after 100 ns of MD simulation, forming stronger interactions. Figure 4H quantifies the changes in the distance between the protein and the centers of mass of the PCB and PEG molecules during the simulation. Within the first 20 ns, the distance between the protein and both PCB and PEG centers of mass decreased continuously, indicating that the surface protein was progressively approaching the polymer surface. Specifically, in the protein-PCB system, the distance from the protein to the PCB center of mass decreased from 5.0 nm to approximately 3.5 nm, while the distance to the PEG center of mass decreased from 4.3 nm to 2.5 nm, reaching a stable state. These results indicate that protein adsorption on the PEG polymer surface occurs more rapidly, resulting in a more stable composite structure.

To elucidate the molecular recognition mechanisms between the protein and the polymers, we investigated the binding modes of the protein to the PCB and PEG surfaces after the MD simulations. First, we examined the hydration layer on the polymer surfaces (Figures 4I-4M). After MD simulation, water molecules completely enveloped the PCB structure, forming a stable hydration layer, while only a tiny portion of the protein structure extended into this layer to interact with the PCB. In the protein-PEG system, however, the hydration layer on the PEG surface was primarily disrupted by the protein, allowing direct interactions between the protein and PEG (Figure 4I). The PCB molecules' O1, O2, and O4 atoms are primarily responsible for hydrogen bonding with water molecules, with an average distance of approximately 0.27 nm (Figure 4J). In the protein-PEG system, the hydroxyl oxygen atoms at the ends of PEG are the primary sites for hydrogen bonding with water. However, their interactions are significantly weaker than those observed in the PCB system (Figure 4K). Consequently, analyzing the radial distribution functions reveals that interactions between PCB and water molecules are considerably stronger than between PEG and water. The binding energies between the polymer and water molecules were further analyzed (Figure 4L). The binding energy of PEG with water molecules exhibited a gradual decrease, likely influenced by protein binding, which results in weakened interactions between PEG and water, with an average value of -4303.47 ± 701.54 kJ/mol. In contrast, the binding energy of PCB with water molecules was significantly more vital, with an average value of -31466.20 ± 500.23 kJ/mol, which suggests that the binding affinity of PCB to proteins may be relatively weaker in comparison.

The variation in the number of water molecules at the PCB and PEG surfaces over the simulation time was further quantified (Figure 4M). The number of surface water molecules in the protein-PCB system remained stable at around 3100, with an average of 3152.02 ± 18.32. In contrast, in the protein-PEG system, the number of surface water molecules decreased progressively during the first 20 ns of simulation, ultimately averaging 2084.21 ± 78.66 after 20 ns. The protein's effect on the reduction of surface water molecules on PEG was nearly one-third, indicating that the protein can disrupt the hydration layer on PEG and interact directly with it.

Next, we studied the interactions between the protein and the polymers (Figures 4N-4T). Charged amino acids such as Asp, Glu, and Arg predominantly bind to the PCB surface, facilitating electrostatic interactions with the positively charged amines and negatively charged carboxyl groups within the PCB structure (Figures 4N and 4O). Overall, only a limited number of amino acids interact with the PCB surface, leading to relatively weak interactions. In contrast, in the protein-PEG system, numerous amino acid residues form hydrogen bonds with PEG (Figures 4P and 4Q). The participating amino acids are widely distributed, including charged, uncharged, and backbone atoms. Thus, it can be inferred that the protein-PEG interaction not only allows for more hydrogen bonds but also results in a substantial surrounding of the protein structure by PEG chains, leading to stronger van der Waals interactions. The binding capabilities of the protein with the PEG surface are greater than those with the PCB surface.

The findings suggest that hydrogen bonding is a crucial driving force for the protein's binding to the polymers 44, 45; hence, we statistically analyzed the changes in hydrogen bond counts throughout the simulation (Figure 4R). During the initial 40 ns, the hydrogen bonding interactions between the protein and PEG gradually strengthened, indicating that the protein adsorbed onto the PEG surface through these interactions. In contrast, hydrogen bonding between the protein and PCB remained weak. After 50 ns, the average hydrogen bond counts in the two systems were 0.32 and 39.09, respectively. To further validate the protein-polymer adsorption, we analyzed the variations in the interaction energies between the protein and PCB (Figure 4S) and between the protein and PEG (Figure 4T) over time. The binding energy between the protein and PCB was approximately zero during the first 20 ns. After this period, the binding energy gradually increased, stabilizing after 70 ns, with average contributions from Coulombic interactions and Lennard-Jones (LJ) interactions of -881.77 ± 52.12 kJ/mol and 22.92 ± 23.30 kJ/mol, respectively. These results indicate that electrostatic interactions are a significant driving force in the PCB's molecular recognition of the protein. The binding energy between PEG and the protein rapidly increased during the first 20 ns. It stabilized after 60 ns, with average contributions from Coulombic and LJ interactions of -1731.14 ± 74.83 kJ/mol and -1371.78 ± 53.75 kJ/mol, respectively. Overall, Coulombic interactions are key drivers in the molecular recognition of the protein by both PCB and PEG, with the interactions between the protein and PEG being significantly stronger than those with PCB. Strong LJ interactions between the protein and PEG further enhance the protein's binding capacity on the PEG surface.

Compared to PEG, PCB forms a more compact and stable hydration layer through stronger interactions with water molecules 46. This hydration layer remains intact against the protein's attack, isolating the polymer from interacting with the protein and contributing to a more efficient resistance to protein adsorption, which is crucial for the prolonged circulation of LNPs in blood 47.

The distribution of siPCSK9-loaded LNPs following intravenous administration was further analyzed by measuring the fluorescence intensity of Cy5-labeled siRNA (Cy5-siRNA) across various organs. This approach allows for a detailed understanding of the biodistribution patterns of the delivered siRNA. Notably, the zwitterionic polymer PCB-modified LNPs demonstrated a significantly enhanced capacity for delivering siPCSK9 to the liver compared to the traditional NeuLNPs. The bio-distribution ratio in the liver reached approximately 60% (Figures 4U and 4V), indicating a preferential accumulation of the therapeutic agent in the target organ. This enhanced liver targeting suggests that PCB-modified LNPs may improve the efficacy of RNAi therapies in treating liver diseases.

### Efficacy of siPCSK9-loaded ZwiLNPs in treating high-fat diet (HFD)-induced hypercholesterolemia in rats

**Figure 5:**
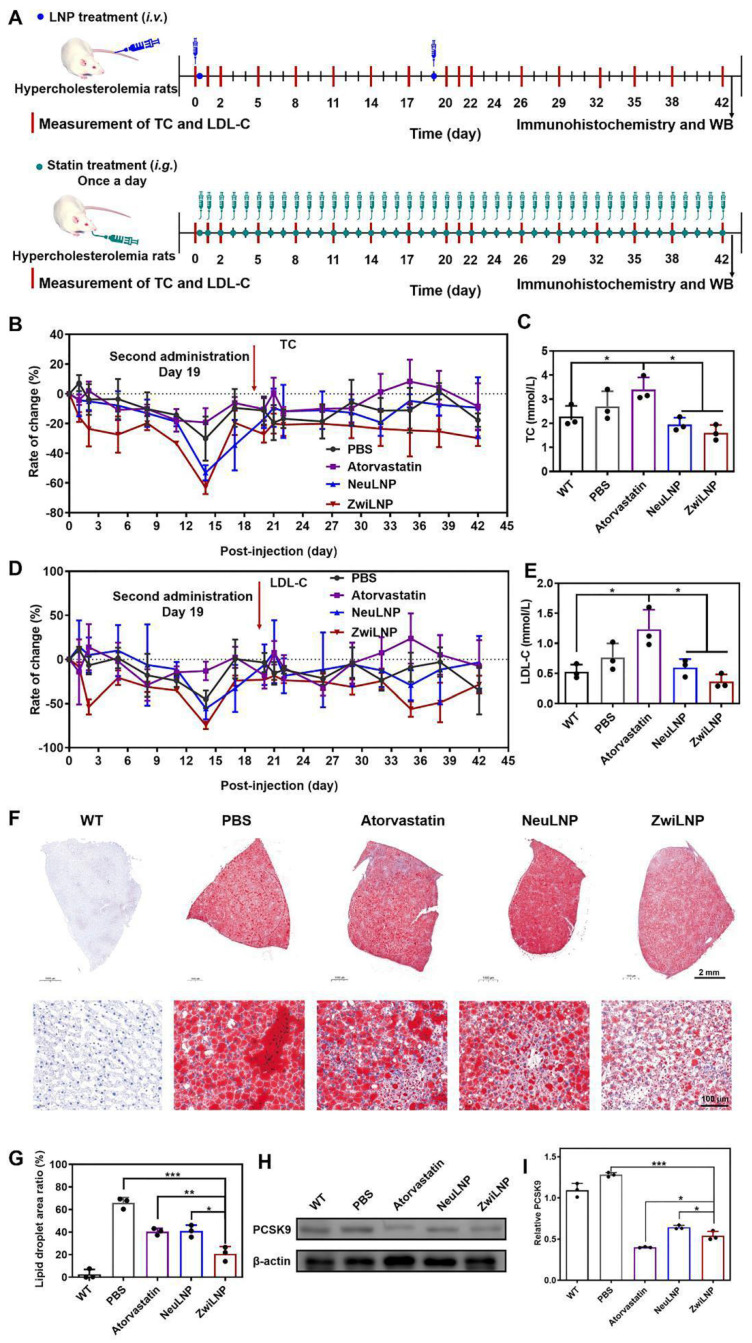
Therapeutic efficacy of ZwiLNPs in HFD-induced rat. (A) The therapeutic schedule of HFD-induced hypercholesteremia rats. (B) Rate of change of TC levels of hypercholesterolemic rats for 42 days. (C) TC levels of hypercholesterolemic rats after treatment. (D) Rate of change of LDL-C levels of hypercholesterolemic rats for 42 days. (E) LDL-C levels of hypercholesterolemic rats after treatment. (F) Oil Red O staining of all groups after treatment. (G) Quantified results of lipid droplet area ratio in Figure 5F using ImageJ. (H) Western blot results of PCSK9 after treatment of all groups. (I) Quantified results of PCSK9 in Figure 5H using ImageJ. Data were presented as the mean ± SD (n = 3). (t-test, *p < 0.05, **p < 0.01, ***p < 0.001)

Compared to small molecule drugs, siRNA therapeutics typically exhibit a longer duration of action, making them promising candidates for treating metabolic disorders. This study evaluated the therapeutic efficacy of siPCSK9-loaded ZwiLNPs in an HFD-induced hypercholesterolemic rat model. Following the administration of LNPs, levels of low-density lipoprotein cholesterol (LDL-C) and total cholesterol (TC) in the rats were continuously monitored to assess the pharmacodynamic response (Figure 5A). Atorvastatin, a well-established cholesterol-lowering agent, was included as a comparative treatment, administered daily due to its relatively short duration of action (Figure 5A).

The results indicated that all LNP formulations exhibited significant efficacy in lowering TC levels, with ZwiLNPs demonstrating the most pronounced reduction. Notably, after 14 days of treatment with LNPs, TC levels in the bloodstream reached their nadir (Figures S14 and 5B), falling below the baseline TC levels observed in normal control rats (Figure 4C). Unexpectedly, treatment with atorvastatin increased TC levels among the HFD-induced hypercholesterolemic rats, suggesting a paradoxical response. By day 17, TC levels in the rats treated with LNPs had significantly increased, prompting a second administration of LNPs on day 19. This subsequent treatment effectively reduced TC levels again.

Throughout the entire treatment regimen, ZwiLNPs consistently lowered TC levels, outperforming NeuLNPs in both initial and subsequent dosing phases (Figure S15). In stark contrast, atorvastatin led to a continuous elevation of TC levels, exacerbating the hypercholesterolemic condition rather than ameliorating it. Additionally, LDL-C levels were monitored as another key outcome measure. The downward trend in LDL-C following treatment with ZwiLNPs and NeuLNPs paralleled the trends observed in TC, underscoring the effectiveness of the LNP treatments (Figures S16, 5D, 5E, and S17). These findings support the potential of ZwiLNPs as a robust therapeutic strategy for managing hyperlipidemia and related metabolic disorders.

After 42 days of treatment, the HFD-induced hypercholesterolemic rats were humanely sacrificed to evaluate the therapeutic outcomes. The livers of these rats were carefully harvested, with specific portions designated for histological sectioning and western blot (WB) analysis. To assess lipid accumulation, liver sections underwent Oil Red O staining, a well-established method for visualizing lipid droplets within tissues (Figure 5F). This staining provided critical insights into the impact of various treatments on hepatic lipid metabolism. The results revealed a significant reduction in lipid droplet accumulation in the livers of rats treated with atorvastatin and LNPs. Among the treatments, ZwiLNPs exhibited the most pronounced effect in lowering lipid droplet levels, indicating their potential effectiveness in mitigating hepatic steatosis associated with hypercholesterolemia. However, despite these improvements, the hepatic lipid content in ZwiLNP-treated rats still showed considerable differences compared to the liver tissues of normal control rats (Figure 5G). This underscores the necessity for ongoing therapeutic strategies to achieve optimal lipid regulation.

Given that LNPs mediate their therapeutic effects by silencing the expression of PCSK9, we further assessed the levels of PCSK9 in the livers of the treated rats through WB analysis (Figure 5H). Interestingly, we observed that atorvastatin also resulted in a notable downregulation of PCSK9 levels. This finding suggests that atorvastatin may exert additional effects beyond its primary mechanism of action, further contributing to its cholesterol-lowering effects. Moreover, the comparative analysis revealed that the RNAi efficacy of ZwiLNPs was significantly superior to that of NeuLNPs (Figure 5I). This highlights the enhanced performance of ZwiLNPs in mediating the silencing of PCSK9, which is crucial for the therapeutic management of hypercholesterolemia.

### Biocompatibility of siPCSK9-loaded ZwiLNPs

**Figure 6:**
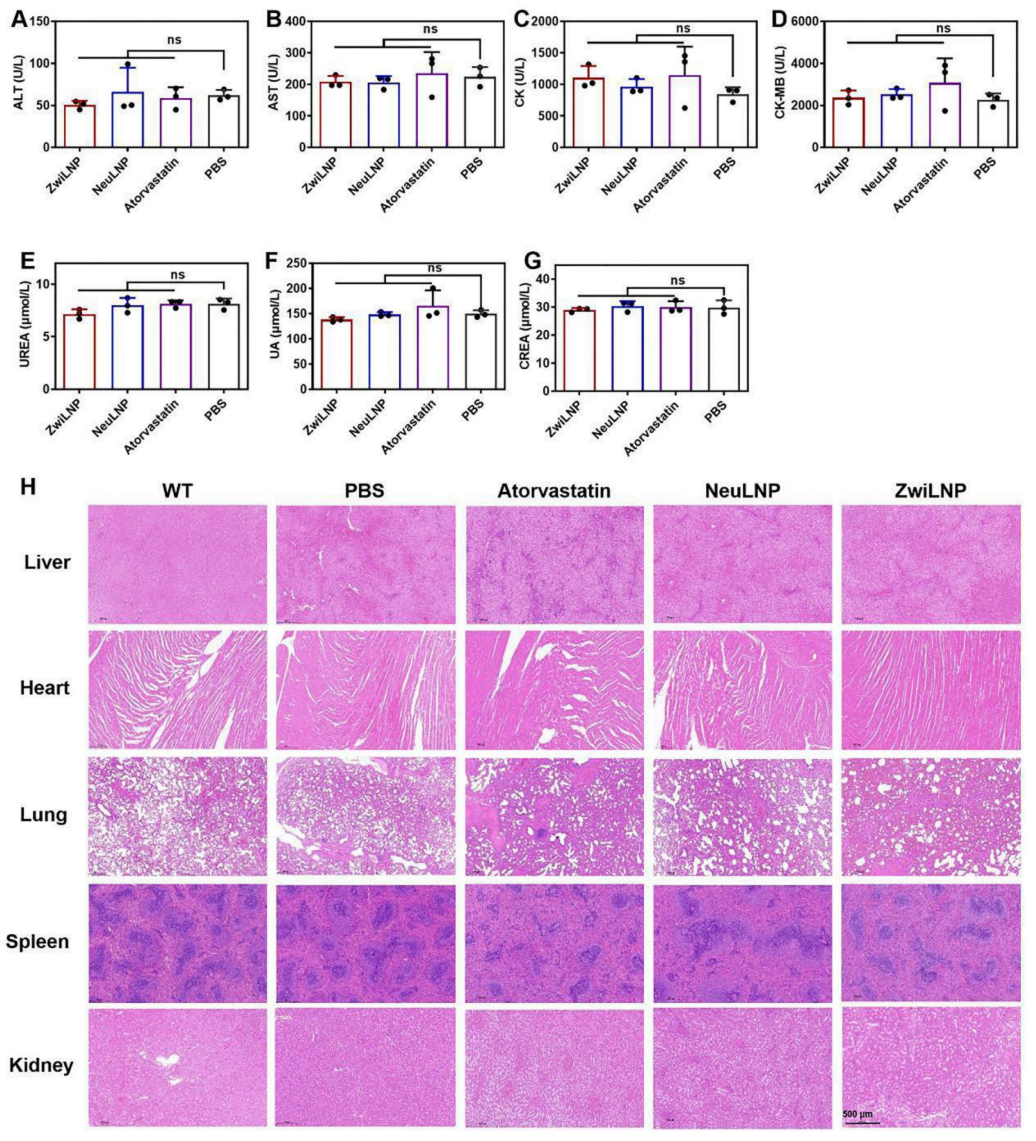
The biocompatibility of ZwiLNPs *in vivo.* (A) ALT levels of rats after treatment. (B) AST levels of rats after treatment. (C) CK levels of rats after treatment. (D) CK-MB levels of rats after treatment. (E) UREA levels of rats after treatment. (F) UA levels of rats after treatment. (G) CREA levels of rats after treatment. (H) H&E staining images of major organs from the treated rat. Scale bar: 500 µm. Data were presented as the mean ± SD (n = 3). (t-test, ns p > 0.05)

We evaluated the biocompatibility of ZwiLNPs through two primary approaches. First, we assessed the potential organ damage caused by ZwiLNPs using biochemical analyses of blood parameters. Following treatment with ZwiLNPs, levels of alanine transaminase (ALT) and aspartic transaminase (AST) did not show significant elevation, indicating that ZwiLNPs do not induce hepatic injury (Figures 6A and 6B). In contrast, some NeuLNPs resulted in increased ALT levels. At the same time, certain doses of atorvastatin were associated with elevated AST levels, suggesting potential hepatic injury risks associated with both NeuLNPs and atorvastatin.

Regarding renal function, both types of LNPs did not cause significant increases in UREA, uric acid (UA), or creatinine (CREA), indicating that neither ZwiLNPs nor NeuLNPs impose a notable burden on the kidneys (Figures 6C-6E). Conversely, some rats treated with atorvastatin exhibited increased UA levels, suggesting a potential risk of renal damage associated with atorvastatin administration.

Regarding myocardial injury, we assessed creatine kinase (CK) and creatine kinase MB (CK-MB) levels (Figures 6F and 6G). Some ZwiLNPs caused a slight elevation in CK levels, indicating a potential risk of myocardial damage. In contrast, NeuLNPs did not result in any significant increases in CK or CK-MB levels. Notably, many rats treated with atorvastatin showed elevated levels of both CK and CK-MB, highlighting a higher risk of myocardial injury associated with atorvastatin treatment. Overall, these results suggest that both types of LNPs are unlikely to cause damage to the liver, kidneys, or heart. In contrast, atorvastatin may pose risks to these organs, particularly concerning myocardial injury, potentially due to the high frequency and dosage of atorvastatin administration.

We also evaluated the biocompatibility of the LNPs and statin medications through histological examination using hematoxylin and eosin (H&E) staining (Figure 6H). No significant pathological changes were observed in the liver, heart, lungs, spleen, or kidneys following treatment with either LNPs or atorvastatin. This finding further corroborates the biocompatibility of the two LNPs and indicates that any myocardial damage caused by atorvastatin is likely minimal.

These results support the conclusion that siPCSK9-loaded ZwiLNPs possess excellent biocompatibility, presenting a promising therapeutic option with a favorable safety profile. In contrast, the potential risks associated with atorvastatin treatment underscore the importance of careful dosage and monitoring in clinical settings.

### Efficacy of sihPCSK9-loaded ZwiLNPs in HFD-induced PCSK9 humanized transgenic mouse model of hypercholesterolemia

We investigated the pharmacodynamics of ZwiLNPs loaded with sihPCSK9 at a lower 1 mg/kg dosage in an HFD-induced PCSK9 humanized transgenic mouse model of hypercholesterolemia. This model is particularly relevant for understanding the efficacy of novel siRNA therapeutics in a setting that closely mimics human metabolic conditions. We also administered Inclisiran, a commercially available targeted PCSK9 siRNA drug, subcutaneously at the same dosage for comparative purposes.

**Figure 7:**
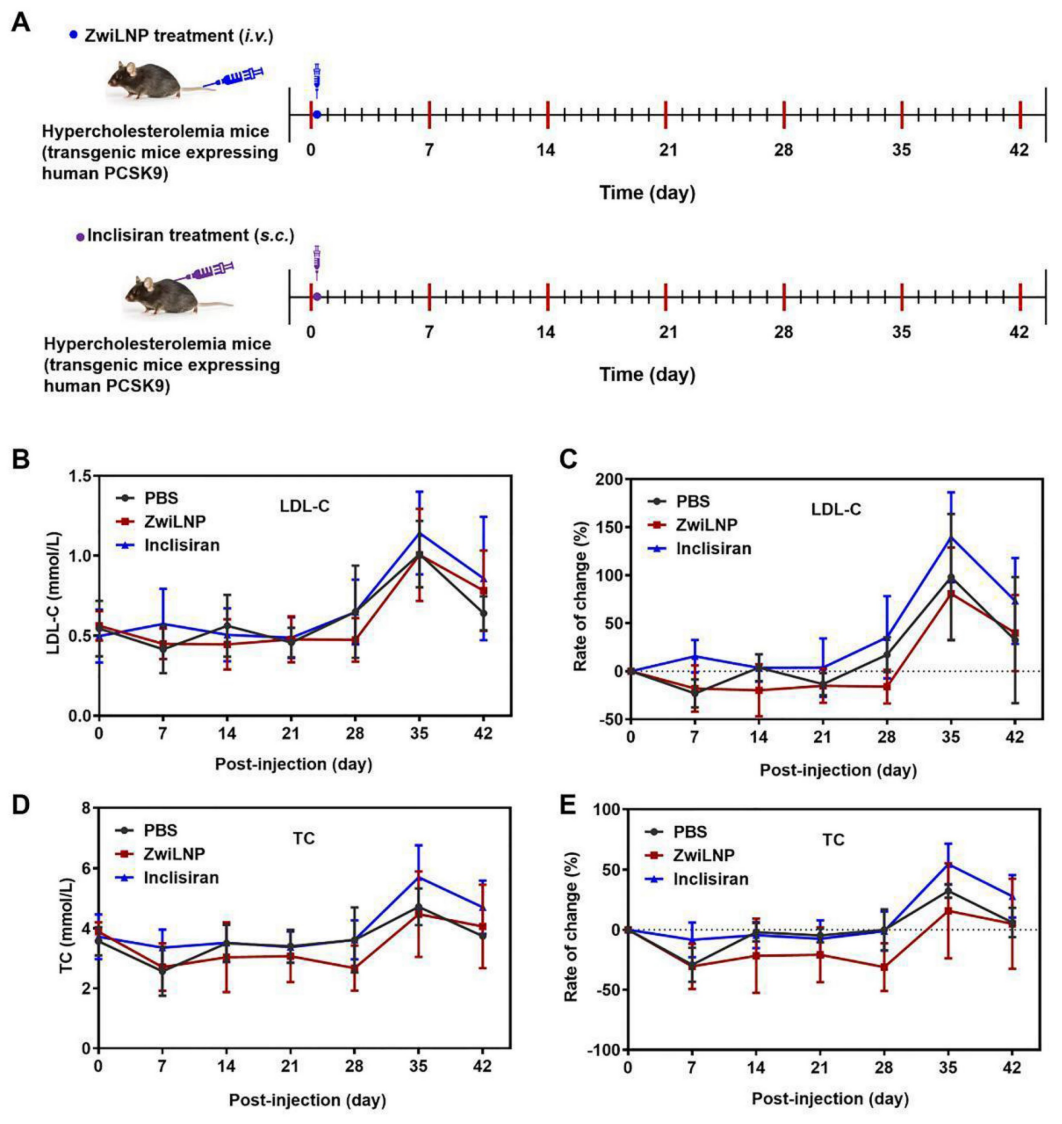
Therapeutic efficacy of ZwiLNPs in HFD-induced PCSK9 humanized transgenic mice. A) The therapeutic schedule of HFD-induced PCSK9 humanized transgenic hypercholesteremia mice. (B) Variation of LDL-C levels of hypercholesterolemic mice for 42 days. (C) Rate of change of LDL-C levels of hypercholesterolemic mice for 42 days. (D) Variation of TC levels of hypercholesterolemic mice for 42 days. (E) Rate of change of TC levels of hypercholesterolemic mice for 42 days. Data were presented as the mean ± SD (n = 3).

Due to constraints on the volume of blood that can be collected from the mice, we measured LDL-C and TC levels every seven days following administration (Figure 7A). The results demonstrated that ZwiLNPs effectively reduced LDL-C (Figures 7B and 7C) and TC (Figures 7D and 7E) levels in the blood. Notably, the reduction in TC levels was more pronounced than that of LDL-C, suggesting a more robust lipid-lowering effect mediated by ZwiLNPs.

As the HFD continued, a significant upregulation of both TC and LDL-C levels was observed in all mice by day 35. Despite a slight decline in these levels by day 42, the values remained substantially elevated, indicating the challenges of managing hypercholesterolemia in sustained high-fat feeding. Importantly, Inclisiran did not exhibit significant downregulation of TC or LDL-C levels throughout the treatment period. This lack of efficacy may be attributed to the lower dosage, highlighting the critical role of dosage in the therapeutic outcomes of siRNA treatments.

The data gathered from this study suggest that ZwiLNPs have a strong potential as a therapeutic strategy for managing hypercholesterolemia, particularly in the context of HFD intake. The observed superior efficacy of ZwiLNPs compared to Inclisiran underscores the need for continued exploration into novel siRNA formulations that enhance delivery and silencing capabilities.

## Discussion

The exploration of ZwiLNPs for siRNA delivery has shown promise as a method for improving the therapeutic management of hypercholesterolemia. Our findings indicate that ZwiLNPs enhance siRNA stability in circulation and improve cellular uptake and endosomal escape compared to conventional lipid nanoparticles (LNPs). These improvements are primarily attributed to zwitterionic lipids' unique charge regulation properties, which allow for a self-modifying charge profile that adapts to environmental conditions. Although these advancements represent meaningful progress in RNA delivery, it is essential to consider the limitations highlighted throughout our study, particularly those relating to the general challenges associated with LNPs and the specific complexities of charge regulation.

One of the notable challenges in the delivery process of LNPs is achieving precise targeting and efficient cellular uptake. While ZwiLNPs demonstrated a superior ability to escape from endosomes, as evidenced by reduced co-localization with LysoTracker™ staining, complete cellular internalization remains a hurdle. LNPs can encounter various biological barriers, including serum proteins that may opsonize the particles, leading to rapid clearance by the mononuclear phagocyte system. This often results in inconsistent dosing and diminished therapeutic efficacy. In our study, significant reductions in serum LDL and hepatic PCSK9 levels were observed, indicating successful target silencing; however, the variability in individual responses across different mouse models suggests that factors like immune response and tissue perfusion require further investigation. Developing strategies to enhance specificity and minimize systemic exposure will be crucial in advancing LNP technologies for more reliable therapeutic outcomes.

Additionally, while the zwitterionic nature of the ZwiLNPs has shown beneficial properties, charge regulation presents significant challenges. The delivery of mRNA and siRNA is heavily influenced by charge interactions between nanoparticles and cellular membranes. The balance is delicate; while cationic lipids can enhance membrane fusion and uptake, excess positive charge can lead to cytotoxic effects and provoke unwanted immune responses. Although we have improved endosomal escape through the proton sponge effect, further optimization in charge tuning remains essential. This necessitates ongoing investigation into the physicochemical properties of LNPs in various biological contexts. Understanding how factors such as pH, ionic strength, and cellular environment influence the charging behavior of LNPs will substantially improve the development of formulations that can navigate physiological barriers more effectively.

Moreover, practical limitations regarding the scalability and reproducibility of LNP formulations are noteworthy. As highlighted in our pharmacokinetic studies, while ZwiLNPs provide an extended circulation half-life, the processes required to consistently manufacture them at scale while ensuring quality and homogeneity are still being developed. Batch-to-batch variability can significantly affect not only the pharmacokinetics of LNPs but also their interaction with biological systems. Ensuring that LNPs can be produced under strict quality controls is paramount for their transition to clinical applications. Regulatory hurdles and the complexities of large-scale production underscore the importance of establishing harmonized manufacturing practices and clearer guidelines to address these challenges.

While ZwiLNPs offer significant advancements in siRNA delivery for hypercholesterolemia, addressing the inherent limitations of LNPs will be critical for their future applications. By focusing on refining charge regulation, enhancing targeting efficiency, and developing robust manufacturing protocols, we can leverage the benefits of LNP technologies to unlock new possibilities for RNA-based therapies. This continued evolution will not only facilitate better patient outcomes but may also broaden the range of diseases that RNA interference strategies can effectively target, paving the way for innovative treatments across a spectrum of conditions.

## Conclusions

In summary, we developed zwitterionic lipid nanoparticles, termed ZwiLNPs, as a novel delivery system for siRNA targeting hypercholesterolemia. ZwiLNPs exhibit a rational charge self-transformation mechanism that enhances biocompatibility and cellular uptake, effectively addressing key challenges in siRNA delivery. Furthermore, ZwiLNPs demonstrate improved circulation times due to a denser hydration layer that reduces protein adsorption and evasion of the immune response. This unique property contributes to their ability to efficiently target liver cells and improve endosomal escape compared to conventional lipid nanoparticles. While our findings are encouraging, further investigations are warranted to refine the formulation and evaluate the long-term safety and efficacy of ZwiLNPs in therapeutic applications. This study adds valuable insights into developing innovative delivery systems for RNA-based therapies.

## Experimental section

### Materials

2-(*N, N'*-dimethylamino) ethyl methacrylate (DMAEMA, 98%) was purchased from Alfa Aesar. β-Propiolactone (98%), copper bromide (98%) and 2-cyanoprop-2-yldithiobenzoate were from J&K Scientific Ltd. *N, N, N', N'', N''*-pentamethyldiethylenetriamine (PMDETA, 99%) and triethylamine (TEA, 99%) were purchased from Sigma Aldrich (St. Louis, Missouri, USA). Cy5-siRNA (sense strand, 5'-UUCUCCGAACGUGUCACGUdTdT-3'; antisense strand, 5'-ACGUGACACGUUCGGAGAAdTdT-3'), negative control siRNA scrambled siRNA (siNonsense) (sense strand, 5'-UUCUCCGAACGUGUCACGUdTdT-3'; antisense strand, 5'-ACGUGACACGUUCGGAGAAdTdT-3') and siRNA against rat PCSK9 (siPCSK9: sense strand, 5'-GCmCmUmGGAGUmUmUmAUmUmCmGGAAdT*dT-3'; antisense strand, 5'-UUCCGAAUmAAACUCCmAGGCdT*dT-3') were synthesized by Suzhou Biosyntech Co., Ltd. (Suzhou, China). Inclisiran (sense strand, 5'-Cm*Um*AmGmAmCmCfUmdTUmUmGmCmUmUmUmUmGmUm-L96-3'; antisense strand, 5'-Am*Cf*AmAfAfAfGmCfAmAfAmAfCmAfGmGfUmCfUmAmGm*Am*Am-3') and siRNA against human PCSK9 (sihPCSK9: sense strand, 5'-Cm*Um*AmGmAmCmCfUmdTUmUmGmCmUmUmUmUmGmUm-3'; antisense strand, 5'-Am*Cf*AmAfAfAfGmCfAmAfAmAfCmAfGmGfUmCfUmAmGm*Am*Am-3') were purchased from Shanghai Primerna biotechnology Co., Ltd. (Shanghai, China). Af = adenine 2'-F ribonucleotide; Cf = cytosine 2'-F ribonucleotide; Gf = guanine 2'-F ribonucleotide; Am = adenine 2'-OMe ribonucleotide; Cm = cytosine 2'-OMe ribonucleotide; Gm = guanine 2'-OMe ribonucleotide; Um = uracil 2'-OMe ribonucleotide; L96 = triantennary N-acetyl-galactosamine; * = sphorothioate linkages. Cholesterol (95%), 1,2-distearoyl-sn-glycero-3-phosphoethanolamine (DSPE), 1,2-Dioleoyl-3-trimethylammonium-propane (DOTAP) and DSPE-PEG were purchased from AVT Pharmaceutical Tech Co., Ltd. (Shanghai, China). Dulbecco's modified eagle medium (DMEM), L-glutamine, penicillin (10,000 U/mL), streptomycin (10 mg/mL), trypsin-EDTA and fetal bovine serum (FBS), LysoTracker Red and Trizol were purchased from Invitrogen (Carlsbad, CA, USA). Paraformaldehyde and 4',6-diamidino-2-phenylindole dihydrochloride (DAPI) were obtained from Solarbio Science & Technology Ltd., Co (Beijing, China). Gel green^TM^, and phosphate-buffered saline (PBS, pH = 7.4) were purchased from the Beyotime Institute of Biotechnology (Nanjing, China). All antibodies used for WB were purchased from eBioscience (CA, USA). All the reagents were used as received without further purification. Other reagents were acquired from Sigma-Aldrich.

### Synthesis and purification of carboxybetaine (CB)

CB monomer was synthesized by the reaction of DMAEMA and β-propiolactone. Specifically, DMAEMA (0.6245 g, 3.97 mmol) was dissolved in ultra-dry dichloromethane and was added to a clean and dry Schlenk flask with a constant-pressure dropping funnel. Oxygen was removed from the system by three freeze-pump-thaw cycles and recharged with nitrogen. β-propiolactone (0.3437 g, 4.77 mmol) was dissolved in ultra-dry dichloromethane and slowly added dropwise to the bottle through a constant pressure funnel at 10 °C, and the reaction was continued to be stirred on crushed ice for 5 h. After the reaction, dichloromethane is removed by rotary evaporation, and an appropriate amount of acetone is added for suction filtration. The resulting product is washed twice with dichloromethane and ether respectively, then dried under vacuum to obtain the final product CB.

### Synthesis and purification of DSPE-Br

DSPE-Br was synthesized by the reaction of DSPE and 2-bromo-2-methylpropionyl bromide. DSPE (0.2972 g, 0.40 mmol) and triethylamine (0.0810 g, 0.80 mmol) were dissolved in ultra-dry dichloromethane and were added to a clean and dry Schlenk flask, followed by stirring at room temperature for a duration of 1 h. 2-bromo-2-methylpropioyl bromide (0.1563 g, 0.68 mmol) was added to the above solution, heated to 45 °C, and condensed for reflux reaction for 10 h. At the end of the reaction, the reaction liquid was transferred into the separation funnel, and after pickling once in turn and deionic water washing 3 times (in which the dichloromethane phase was in the lower layer of the separation funnel), the organic phase was dehydrated with anhydrous Na_2_SO_4_, and the solid Na_2_SO_4_ was removed by filtration. A rotary evaporator removed the solvent dichloromethane and the white solid was obtained.

### Synthesis and purification of DSPE-PCB_n_

DSPE-PCB with the theoretical DP of 20 was synthesized according to the method reported in our laboratory using an atom transfer radical polymerization (ATRP). DSPE-Br (0.0358 g, 0.04 mmol), CB (0.0358 g, 0.04 mmol), and Cu(Ⅰ)Br (0.0358 g, 0.04 mmol) were dissolved in dichloromethane and ethanol, and were added to a clean and dry Schenk flask. The system was degassed by three freeze-pump-thaw cycles and recharged with nitrogen. Then, PMDETA (0.0139 g, 0.08 mmol) was dissolved in ethanol and added to the reaction mixture. The system was degassed by three freeze-pump-thaw cycles again, recharged with nitrogen, and stirred for 24 h at 60 ℃. The impurities and unreacted monomers were removed by dialyzing in a Cellu SepH1-membrane (MWCO 3,500) against dichloromethane, ethanol, and deionized water for 48 h, respectively. The final product was obtained by lyophilization.

### Preparation and characterization of cationic liposomes and their complexes

Cationic liposomes were prepared by thin film dispersion method, with DSPE-PCB_16_, cationic lipid molecules DOTAP and cholesterol, according to molar ratio (1:1:0.3). Specifically, cationic lipid DOTAP (5.25 mg, 0.015 mmol), cholesterol (2.9 mg, 0.015 mmol) and DSPE-PCB16 (9.4 mg, 0.006 mmol) were dissolved in dichloromethane and ethanol, and were added into 100mL nightshade bottle. The solvent was removed and lipid film was formed by rotary evaporation apparatus at 140 rpm and 45 °C. Then, the nightshade bottle was placed in a vacuum-drying oven for 2 h to ensure that all solvents were removed. 1.5 mL of lemon-sodium citrate buffer solution (pH = 4) was added to the flask. Using a digitally controlled ultrasonic cleaner at a frequency of 90%, ultrasonication was carried out for 30 min to form a translucent emulsion. Employing the same preparation method, cationic liposomes with molar ratios of (1:1:0.4), (1:1:0.2), and (1:1:0.1) were fabricated. DSPE-PEG 2000 (0.006 mmol) was used to substitute the DSPE-PCB lipid molecules to prepare DSPE-PEG 2000-modified cationic liposomes. The cationic liposomes were mixed with siRNA (dissolve in DEPC-treated water) by the corresponding mass ratio and left to stand at room temperature for 30 min to obtain the cationic liposome/siRNA complex.

The morphological analysis was carried out by TEM (JEM-1200EX). The zeta potential of particles was characterized with a Malvern Zetasize Nano ZS instrument.

### The siRNA binding ability, encapsulation rate and drug loading rate of LNPs

The siRNA binding ability of LNPs was evaluated by agarose gel retardation assay. LNPs were prepared at various N/P ratios, and naked siRNA was used as a control. 10 μL of LNPs were mixed with 2 μL of 6×loading buffer, and then the mixture was loaded onto 2% (w/v) agarose gel containing Gel Red^TM^ nucleic acid stain. Electrophoresis was performed at a constant voltage of 110 V for 10 min in a 1×TAE running buffer. The migration of siRNA bands was visualized and photographed with a UV Transilluminator at a wavelength of 254 nm.

The encapsulation rate and loading rate of siRNA in LNPs were calculated using the equations listed below, respectively.

Encapsulation rate (%) = W_siRNA in LNP_ / W_total siRNA_ ×100%

Loading rate (%) = W_siRNA in LNP_ / W_total LNP_ ×100%

### The serum stability of LNPs

All groups of LNPs were added into the DMEM with 10% FBS. After incubated for different time, the samples were measured by DLS.

### Proton buffering capability of LNPs

The proton buffering capacity of LNPs was detected by acid-base titration.

### MD simulations

The MD simulations were performed using the Gromacs 2019.6 program 48, under constant temperature and pressure conditions with periodic boundary conditions. The Amber14SB all-atom force field 49 and the TIP3P water model 50 were employed. During the simulations, hydrogen bonds were constrained using the LINCS algorithm 51, with an integration timestep of 2 fs. Electrostatic interactions were computed using the Particle Mesh Ewald (PME) method 52. Non-bonded interactions were truncated at 10 Å, with updates occurring every 10 steps. The V-rescale method 53 was used for temperature coupling, maintaining the system at 300 K, while pressure was controlled at 1 bar using the Parrinello-Rahman algorithm 54. Initially, energy minimization was performed on both systems using the steepest descent method to eliminate any close atomic contacts. This was followed by a 100 ps NVT equilibration at 300 K. Finally, two separate 100 ns production runs were carried out for the two systems, with configurations saved every 20 ps. Visualization of the simulation results was performed using the embedded tools in Gromacs and VMD.

### Cell culture

HepG2 cells were cultured in DMEM supplemented with 10% FBS and 1% penicillin/streptomycin. They were incubated at 37 °C in 5% CO_2_.

### Cell culture and endosomal escape

The intracellular trafficking of various formulations with Cy5-siRNA was investigated using a CLSM. In brief, HepG2cells were seeded in Petri dishes at 2×10^5^ cells/well and allowed to attach for 24 h. The growth medium was then removed and replaced with 500 μL fresh culture medium containing different formulations, which involved LNPs including 1 μg Cy5-siRNA. After 3 h and 5h incubation, the cells were rinsed three times with 1×PBS followed by staining with LysoTracker Red for 30 min at 37 ℃. The cells were washed three times with 1×PBS and fixed in 4% paraformaldehyde for 10 min at room temperature. The nuclei were stained with DAPI for 10 min at room temperature. The cells were observed using a Zeiss LSM780 confocal microscopy (Zeiss Co., Germany).

### Biodistribution and pharmacokinetics

All animal experiments in this article were carried out in accordance with guidelines evaluated and approved by Institutional Animal Care and Use Committee (IACUC-AMSS-20211106-01).

To preliminarily explore the distribution and residence time of the drug *in vivo* after tail vein administration, the lipid-soluble Cy5 dye was loaded in the LNPs. The drug (10 μg Cy5-siRNA/mice) was injected into the mice through the tail vein, after 4-6 h, mice were sacrificed various organs (heart, liver, spleen, lungs, kidneys) were dissected out and the Cy5 fluorescence distribution and intensity were detected by the Kadak *in vivo* imaging system. The organs were extracted, and methanol was added to grind them under dark conditions. The solution was vortexed and immediately centrifuged at 14000 rpm at 4 °C for 10 min. The concentration of Cy5 fluorescence in the supernatant was measured with a multimode reader.

To determine the pharmacokinetics of the LNPs, Balb/c mice (male) were randomly divided into three groups. The pharmacokinetic detection was conducted after two administrations, with an interval of three days between the two administrations.

Both the first administration and the second administration were conducted under the following conditions:

(1) Control group: 200 μL of PBS (pH = 7.4) as the control group;

(2) DSPE-PCB_16_ Group: Prepare DSPE-PCB_16_ cationic liposomes loaded with Cy5 fluorescent molecules at a concentration of 2 mg/mL, and take 300μL/per dose of the corresponding mass of siRNA (1μg/μL);

(3) DSPE-PEG 2000 Group: The method is the same as above.

At different time points (0.5, 1, 3, 6, 8, 12, 24 h), 200 μL of blood were collected by eye puncture. 100 μL DMSO was mixed with the blood and the mixture was transferred to a 96-well plate. The fluorescence intensity of the sample was measured by a plate reader at λ_ex_: 625 nm and λ_em_: 670 nm.

### The establishment of HFD-induced hypercholesterolemia rats

To establish an HFD-induced hypercholesterolemia model, thirty-five SD rats (5-week-old, male) were selected and subjected to adaptive feeding for one week. They were randomly divided into five groups (WT, PBS, atorvastatin group, NeuLNPs, and ZwiLNPs). Five rats in the blank control group were fed with normal feed, while the remaining 30 rats were fed with high-fat feed for 3 to 4 weeks.

After 3 to 4 weeks of HFD feeding, serum was collected from the orbital venous plexus of the rats and centrifuged at 4 °C and 4,000 rpm for 10 min. The supernatant was obtained and the contents of serum TC and LDL-C were detected using a biochemical analyzer. The model group was compared with the normal group based on the existing normal ranges of the four lipid parameters in serum (TC: 1.17~3.13, LDL: 0.23~0.75). Rats with significantly elevated levels of TC, TG, and LDL-C, decreased HDL-C content, and significantly higher body weight than the normal group was selected, indicating successful model establishment. From the successfully modeled rats, five groups were randomly divided (WT, PBS, atorvastatin group, NeuLNPs, and ZwiLNPs), with 3 to 5 rats in each group. The establishment of the B6-PCSK9 humanized transgenic mouse model is the same as above.

### Lipid-lowing effects of LNPs in HFD-induced model

After the successful establishment of the model, the four lipid parameters in the serum of 14 model rats and 3 normal rats are detected before drug administration. HFD-induced animals were divided into three groups. On days 0 and 18, two groups of them were treated with NeuLNPs, and ZwiLNPs, each at an equivalent 0.59 mg/kg siPCSK9. One group of them was administered atorvastatin calcium tablets orally with a dose of 5mg/kg/day, lasting for 3 weeks. Normal animals were fed a standard chow diet and HFD-induced animals were HFD during the whole study.

After administration, the blood lipid four items and plasma were detected in rats every 3 days. The weight, liver function, cardiac enzymes, and renal function (ALT, AST, CK, CK-MB, CREA, UREA, and UA) were detected every 14 days. On day 42, all rats were anesthetized. The whole blood and serum were collected. The liver tissues were isolated and then imaged. The liver, spleen, heart, lung, and kidneys were isolated and fixed in 4% formalin for H&E staining. Furthermore, the liver samples were also stained with oil red O to determine the level of lipid accumulation. Levels of aspartic AST, ALT, TC, and LDL-C were detected using serum biochemistry analysis. Additionally, the relative protein expression of PCSK9 was determined using WB. A complete blood specimen was used for serum chemistry analysis and routine blood count, and the serum biochemical indicators were analyzed using Mindray BS-420 automatic biochemistry analyzer.

### Statistical analysis

All data were expressed as mean ± SD. Statistical significance was analyzed using a two-tailed t-test (ns p> 0.05, *p < 0.05, **p < 0.01, ***p < 0.001). Statistical analysis was carried out using GraphPad Prism 9.5.1 software. The electropherograms and WB images were quantified by ImageJ software. Confocal images were quantified by Image-Pro Plus software.

## Supplementary Material

Supplementary figures.

## Abbreviations

siRNA: small interfering RNA
PCSK9: proprotein convertase subtilisin/kexin type 9
LDL: low-density lipoprotein
LNPs: lipid nanoparticles
ZwiLipo: lipid nanoparticles containing zwitterionic polymers
ZwiLNPs: zwitterionic lipid nanoparticles
TEM: transmission electron microscopy
DLS: dynamic light scattering
NeuLNPs: neutral lipid nanoparticles
CLSM: confocal laser scanning microscopy
RNAi: RNA interference
PEG: polyethylene glycol
ABC: accelerated blood clearance
MD: molecular dynamics
RMSD: root mean square deviation
Rg: radius of gyration
SASA: solvent-accessible surface area
LJ: Lennard-Jones
HFD: high-fat diet
LDL-C: low-density lipoprotein cholesterol
TC: total cholesterol
ALT: alanine transaminase
AST: aspartic transaminase
UA: uric acid
CREA: creatinine
CK: creatine kinase
CK-MB: creatine kinase MB
WB: western blot
DMAEMA: 2-(N,N'-dimethylamino) ethyl methacrylate
PMDETA: N,N,N',N'',N''-pentamethyldiethylenetriamine
TEA: triethylamine
Cy5-siRNA: Cy5-labeled siRNA
siNonsense: negative control siRNA scrambled siRNA
siPCSK9: siRNA against rat PCSK9
sihPCSK9: siRNA against human PCSK9
DSPE: 1,2-distearoyl-sn-glycero-3-phosphoethanolamine
DOTAP: 1,2-Dioleoyl-3-trimethylammonium-propane
DSPE-PEG: 1, 2-distearoyl-sn-glycero-3-phosphoethanolamine-N-[methoxy (polyethylene glycol)-2000]
DMEM: Dulbecco's modified eagle medium
FBS: fetal bovine serum
PBS: phosphate-buffered saline
CB: carboxybetaine
ATRP: atom transfer radical polymerization
PME: Particle Mesh Ewald
H&E: hematoxylin and eosin
AST: aspartic transaminase
ALT: alanine transaminase
