# Supplementary material for: Zwitterionic lipid nanoparticles for efficient siRNA delivery and hypercholesterolemia therapy with rational charge self-transformation
Source: Theranostics. 2025 Mar 1;15(8):3693–712. doi: 10.7150/thno.111685 (PMC11905131; doi:10.7150/thno.111685)
Supplement: Supplementary file 1 — Supplementary figures. [file thnov15p3693s1.pdf]

Zwitterionic lipid nanoparticles for efficient siRNA delivery and hypercholesterolemia therapy with rational charge self-transformation

Ruichen Zhao[1,4,#], Jing Guo[1,4,#], Ziqi Liu[1,#], Yusheng Zhang[3], Jiamin Zuo[1,4,], Songzhang Lv[1,4], Xianyu Li[3,*], Wenlong Yao[2,*], Xin Zhang[1,4,*]

1. State Key Laboratory of Biopharmaceutical Preparation and Delivery, Chinese Academy of Sciences, Beijing, 100190, PR China
2. State Key Laboratory of Natural and Biomimetic Drugs, School of Pharmaceutical Sciences, and Chemical Biology Center, Peking University, Beijing, 100191, PR China
3. Beijing Key Laboratory of Traditional Chinese Medicine Basic Research on Prevention and Treatment for Major Diseases, Experimental Research Center, China Academy of Chinese Medical Sciences, Beijing, 100700, PR China
4. School of Chemical Engineering, University of Chinese Academy of Sciences, Beijing, 100049, PR China

This PDF file includes:

1. Supplemental Figure S1-S17

Supplemental Figures

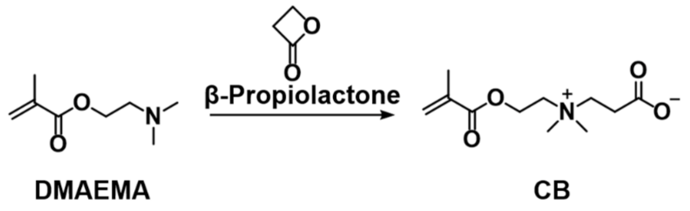

Figure S1. The synthetic route of CB.

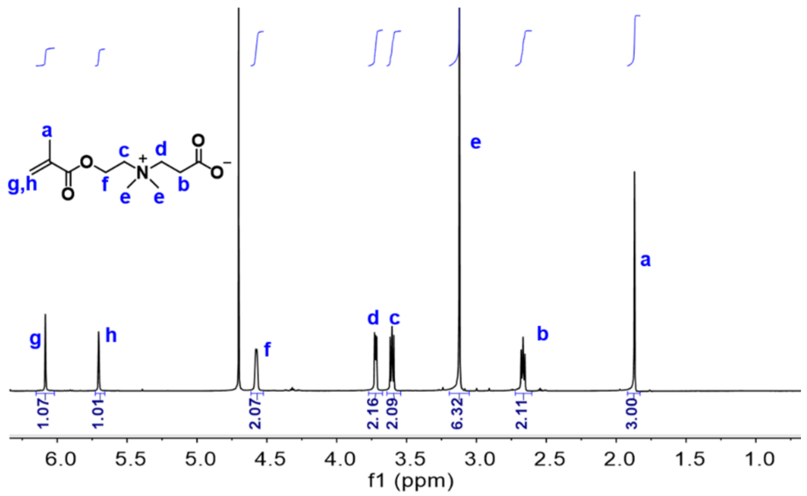

Figure S2. The ^{1}H NMR spectrum of CB.

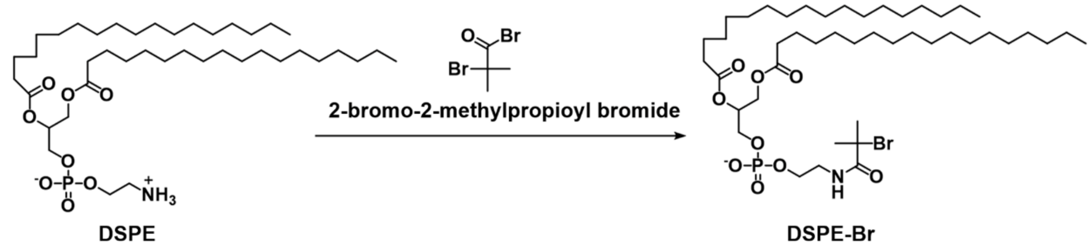

Figure S3. The synthetic route of DSPE-Br.

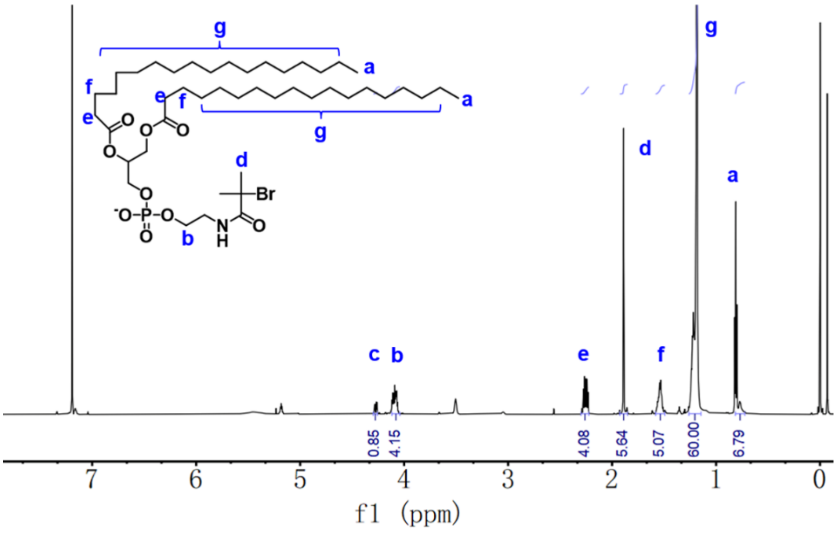

Figure S4. The ^{1}H NMR spectrum of DSPE-Br.

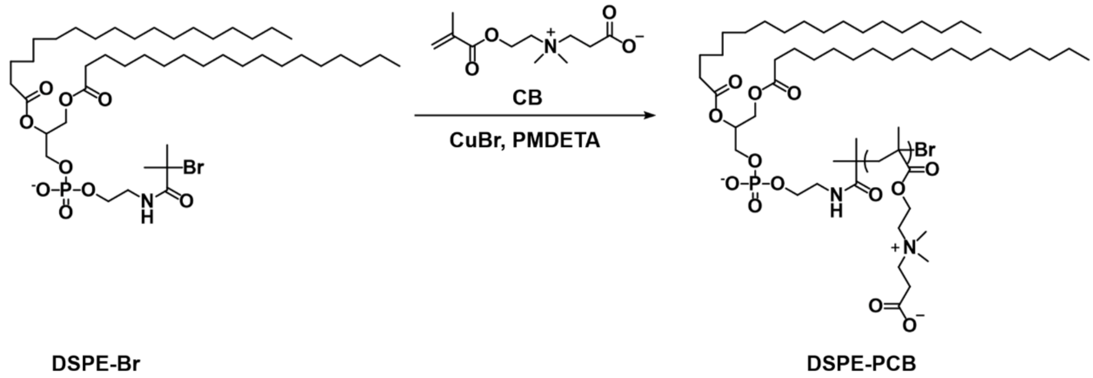

Figure S5. The synthetic route of DSPE-PCB.

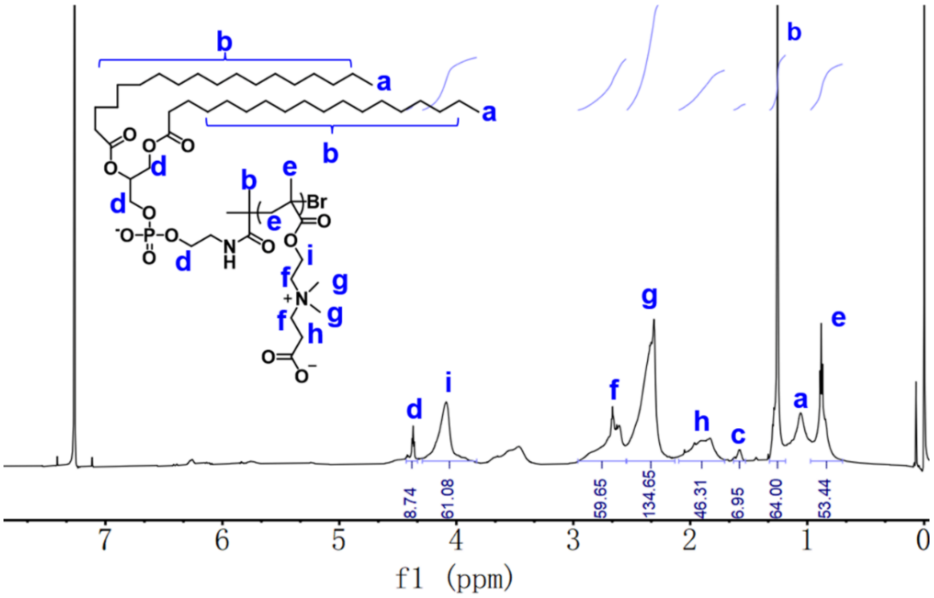

Figure S6. The ^{1}H NMR spectrum of DSPE-PCB.

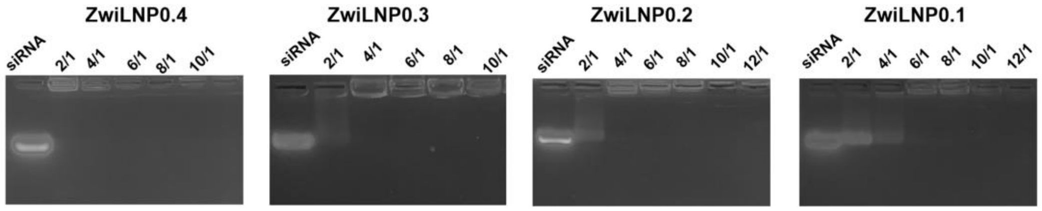

Figure S7. The images of agarose gel electrophoresis of ZwiLNPs.

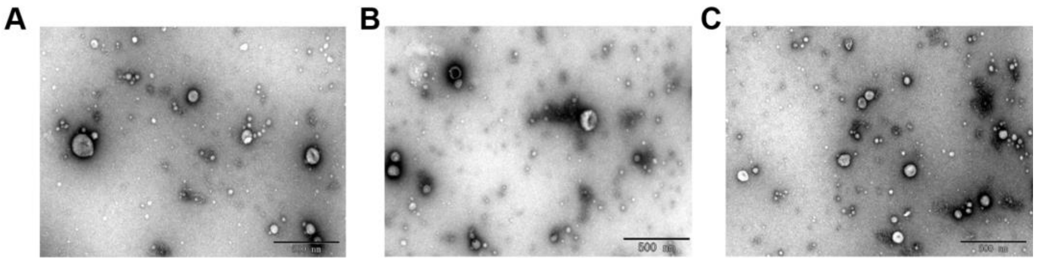

Figure S8. The transmission electron microscopy (TEM) images of ZwiLNPs. (A) ZwiLNP0.2. (B) ZwiLNP0.3. (C) ZwiLNP0.4. Scale bar: 500 nm.

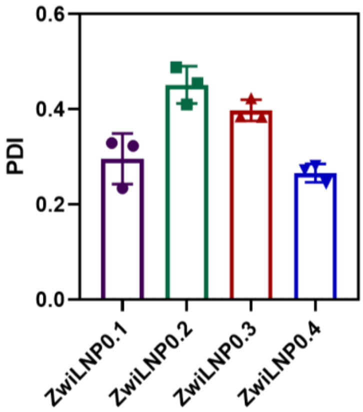

Figure S9. The PDI of ZwiLNP 0.1-0.4. Data were presented as the mean ± SD (n = 3).

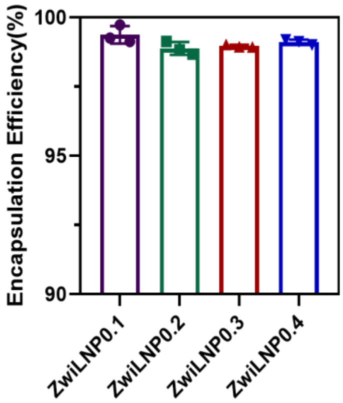

Figure S10. The encapsulation efficiency of ZwiLNP 0.1-0.4. Data were presented as the mean ± SD (n = 3).

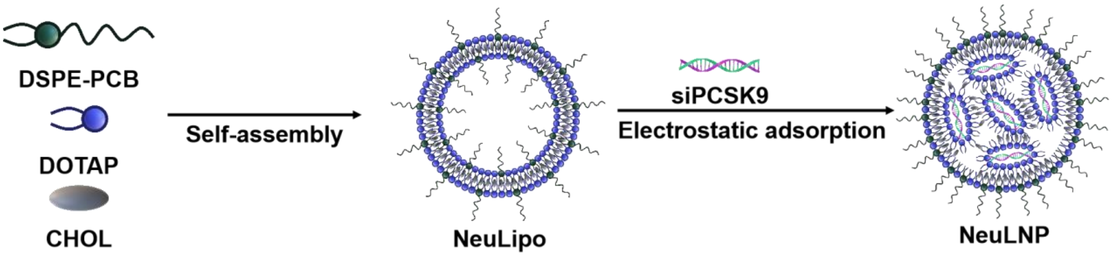

Figure S11. The composition of NeuLNP.

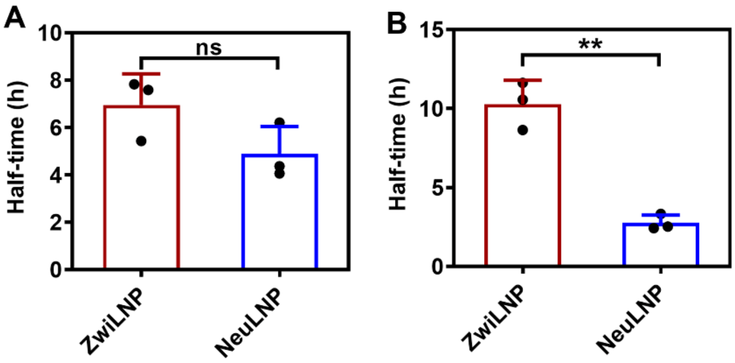

Figure S12. The half-time of ZwiLNPs and NeuLNPs after the first administration (A) and the second administration (B). Data were presented as the mean ± SD (n = 3). (t-test, ns $p>0.05$, ** $p < 0.01$)

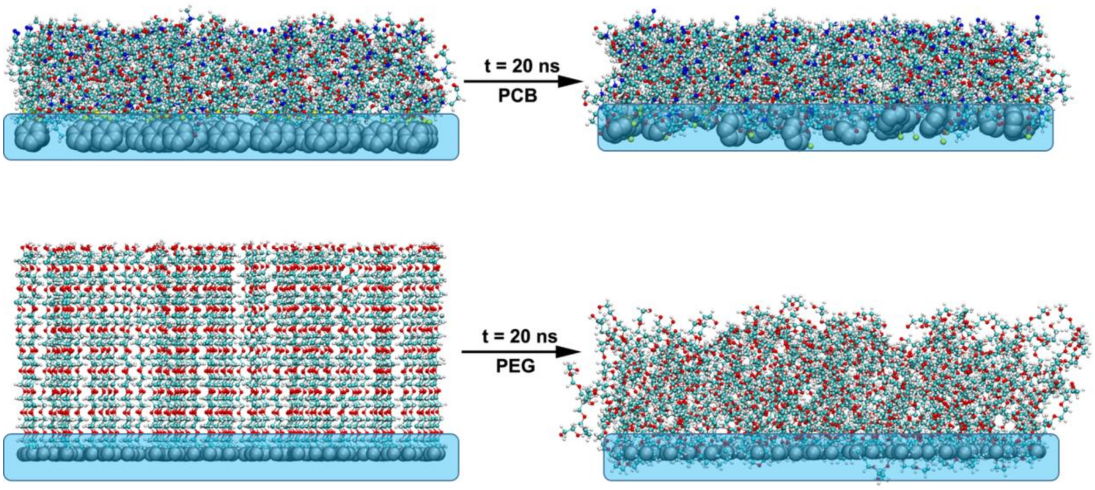

Figure S13. The conformational changes of the PCB and PEG before and after the simulation (the atoms fixed during the simulation are shown in light blue).

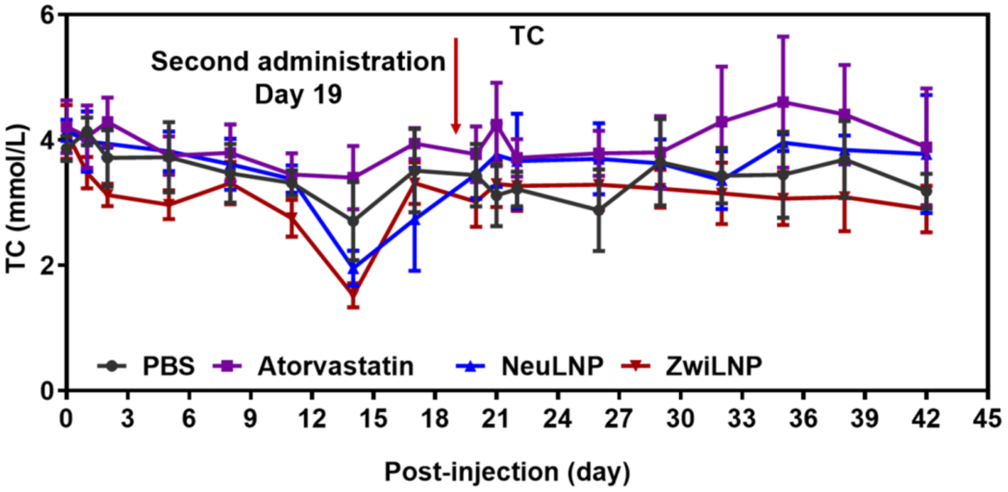

Figure S14. Variation of TC levels of hypercholesterolemic rats for 42 days. Data were presented as the mean ± SD (n = 3).

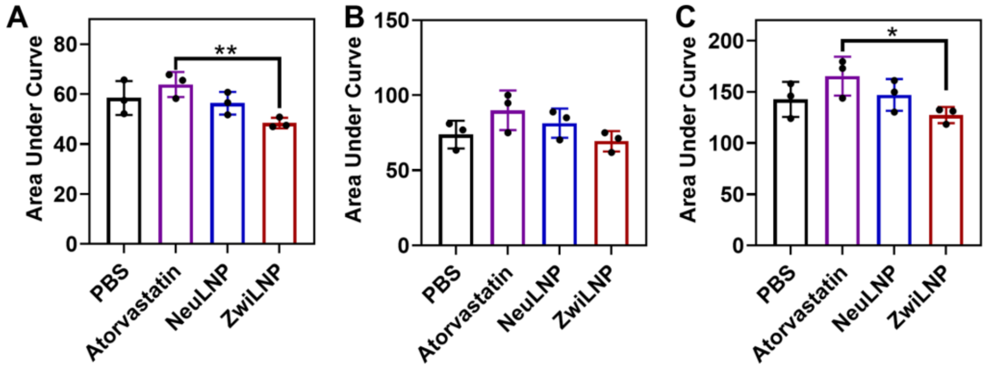

Figure S15. Area under the curve of TC levels of hypercholesterolemic rats for different time periods. (A) Day 0-day 18. (B) Day 19-day 42. (C) Day 0-day 42. Data were presented as the mean ± SD (n = 3). (t-test, *p < 0.05, **p < 0.01)

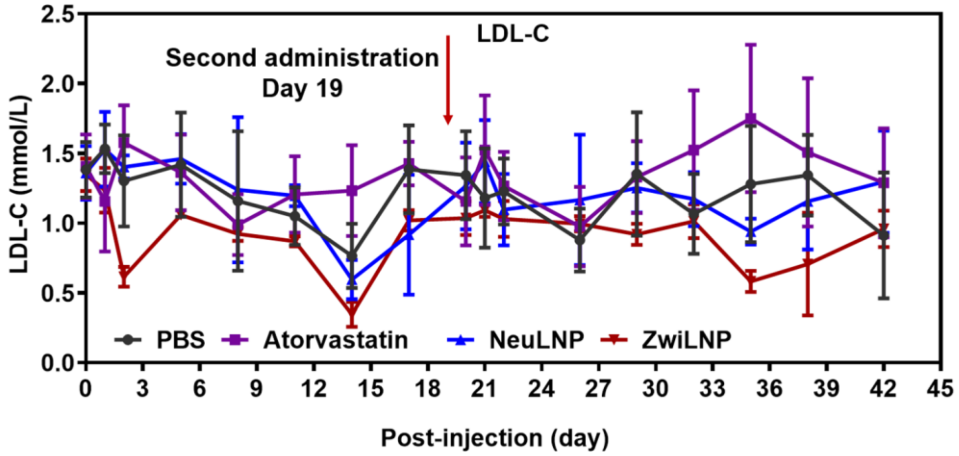

Figure S16. Variation of LDL-C levels of hypercholesterolemic rats for 42 days. Data were presented as the mean ± SD (n = 3).

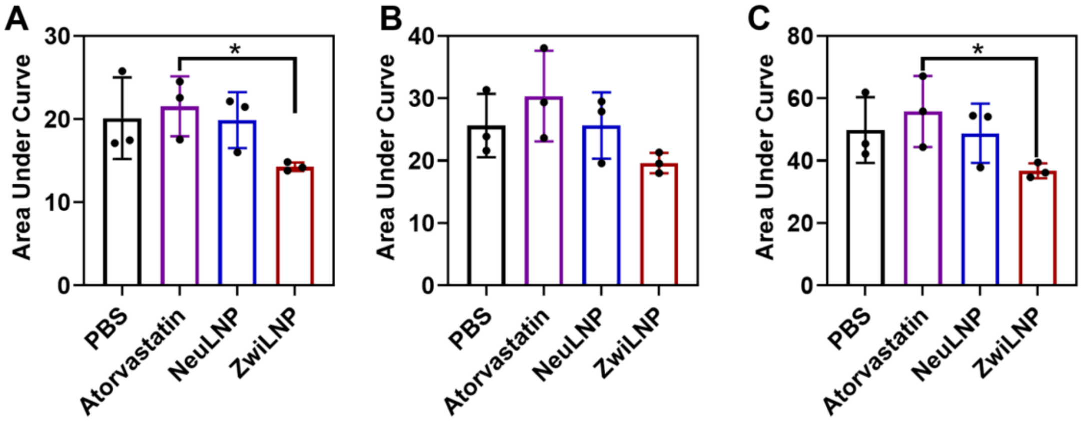

Figure S17. Area under the curve of LDL-C levels of hypercholesterolemic rats for different time periods. (A) Day 0-day 18. (B) Day 19-day 42. (C) Day 0-day 42. Data were presented as the mean ± SD (n = 3). (t-test, *p < 0.05)
